# Contextualizing family planning messages for the BornFyne-PNMS digital platform in Cameroon: a community-based approach

**DOI:** 10.1186/s12978-024-01842-w

**Published:** 2024-08-26

**Authors:** Miriam Nkangu, Sarah Pascale Ngassa Detchaptche, Mildred Njoache, Arone Fantaye, Franck Wanda, Valery Ngo, Pamela Obegu, Mwenya Kasonde, Amos Buh, Regina Sinsai, Evrard Kepgang, Odette Kibu, Armel Tassegning, Nkengfac Fobellah, Nfongue Elate, Alice Tabebot, Donald Weledji, Julian Little, Sanni Yaya

**Affiliations:** 1 Health Promotion Alliance of Cameroon (HPAC), Yaounde, Cameroon; 2 Bruyere Research Institute, Ottawa, Canada; 3 School of Epidemiology and Public Health, University of Ottawa, Ottawa, Canada; 4 Faculty of Medicine, University of Ottawa, Ottawa, Canada; 5 The International Centre for Research and Education and Care (CIRES), Akonolinga, Cameroon; 6 Nkafu Policy Institute, Denis and Lenora Foretia Foundation, Simbock, Yaounde, Cameroon; 7 Liverpool School of Tropical Medicine, Liverpool, UK; 8 Interdisciplinary School of Health Sciences, University of Ottawa, Ottawa, Canada; 9 University of Buea, Buea, Cameroon; 10 Ministry of Public Health Cameroon, Yaounde, Cameroon; 11 Association Camerounaise Pour Le Marketing Social(ACMS), Yaounde, Cameroon; 12 SPRL Donwel Systems, Bruseels, Belgium; 13 School of International Development and Global Studies, University of Ottawa, Ottawa, Canada

**Keywords:** Family planning, Content contextualization, Digital health, Community health care, Pregnancy, Contraceptives

## Abstract

**Background:**

Family planning (FP) is crucial for reducing maternal and infant mortality and morbidity, particularly through the prevention of unsafe abortions resulting from unwanted pregnancies. Despite Cameroon’s commitment to increasing the adoption of modern FP strategies, rural and poor populations still exhibit low demand due to limited access to healthcare services. This study documents the approach in developing family planning messages for the BornFyne prenatal management system as a platform to improve family planning awareness and enhance uptake.

**Method:**

This is a mixed-methods study that employed the Health Belief Model (HBM). The study included a cross-sectional survey and focus group discussions in four districts of Cameroon. The survey explored household perspectives of FP and the use of mobile phone. Focus group discussions involved women, men, and community health workers to gain in-depth insights. Thematic analysis using themes from the HBM guided the analysis, focusing on perceived benefits, barriers, and cues to action.

**Results:**

The survey included 3,288 responses. Thematic analysis of focus group discussions highlighted knowledge gaps and areas requiring additional information. Identified gaps informed the development of targeted FP messages aligned with BornFyne objectives and the Health Belief Model. Results revealed that most respondents recognized the benefits of FP but faced knowledge barriers related to side effects, cultural influences, and communication challenges between partners. Focus group discussions further highlighted the need for education targeting both men and women, dispelling misconceptions, and addressing adolescent and youths’ ignorance. The study emphasized the importance of tailored messaging for specific demographic groups and culture.

**Conclusion:**

Developing effective FP intervention messages requires a nuanced understanding of community perspectives. The BornFyne-PNMS family planning feature, informed by the Health Belief Model, addresses knowledge gaps by delivering educational messages in local dialects via mobile phones. The study’s findings underscore the importance of community-based approaches to contextualizing and developing FP content targeting specific populations to generate tailored messages to promote awareness, acceptance, and informed decision-making. The contextualized and validated messages are uploaded into the BornFyne-family planning feature.

**Supplementary Information:**

The online version contains supplementary material available at 10.1186/s12978-024-01842-w.

## Background

Family planning (FP) is an effective intervention for reducing maternal and infant mortality and morbidity [1, 2]. Unsafe abortions, which often are a result of unwanted pregnancy, are a leading cause of maternal mortality [3]. FP strategies can effectively reduce unwanted pregnancies and, in turn, reduce the need for unsafe abortion [3]. FP enables adolescents, who are at greater risk of experiencing maternal health complications and death during early motherhood, to delay pregnancy [3]. Research has also found that limiting the number of pregnancies, especially among older women, can reduce the risk of maternal mortality [3]. Lengthening the interval between births and reducing poorly timed pregnancies among adolescents, and pregnancies among women who have passed the ideal childbearing age have been found to reduce infant mortality [3, 4]. Other benefits of FP include the prevention of sexually transmitted infections and sustainable population growth, as well as enhanced education and economic empowerment for women [3–7].

Although Cameroon remains committed to increasing the adoption of modern FP strategies among the population, the demand for FP remains low among rural and poor populations [8]. Women in these areas are less exposed to, and less informed about FP due to poor access to healthcare services [7–9]. Compared to urban and wealthy women, women in rural and poor areas are 30% and 50% more likely to suffer maternal death compared to urban and rich women, respectively [9]. Women most often become familiar with FP issues during antenatal and postpartum clinic visits; however, some women are unable to adequately access these services, partly due to high out-of-pocket expenses [9, 10]. In addition, most of the women in rural areas are farmers with limited education and are usually not able to be at home often to listen to radio broadcasts of public health messages about FP [9]. This disadvantage is further compounded by the fact that some confessional healthcare facilities do not provide FP services.

According to data from the FP2030 strategic agenda for Cameroon, a significant portion of women of childbearing age in Cameroon, particularly adolescents and young women, have an unmet need for modern contraceptive [9, 11, 12], with rates reaching 31% among those aged 15–19 and 35% among those aged 20–24 [11]. A considerable proportion (11%) of women of childbearing age are postpartum and not utilizing modern contraception, presenting a prime opportunity for investments in postpartum family planning to boost modern contraceptive prevalence rate [11]. Awareness of modern contraceptive methods is relatively high, with 96.3% in rural areas and 98.6% in urban areas, and 96.9% of women and 99.4% of men aged 15–49 in a union being aware of at least one method [9, 11, 12]. However, family planning needs are less met in rural areas compared to urban areas, with 34.7% versus 50.1%, respectively [7, 11]. Moreover, modern contraceptive usage is lower in rural areas, with 10.6% of women aged 15–49 in a union using modern contraceptive compared to 20.7% in urban areas [9, 11, 12].

A recent study on FP in the northwest region of Cameroon indicated that adoption of FP contraceptive increased in areas targeted for performance-based financing (PBF) interventions compared to control areas [9]. As part of PBF intervention, residents received home visits that enabled households that had been unexposed to FP radio messages to receive FP advice from community health workers through home visits (CHWs) [9]. A study in Kenya indicates SMS interventional messages improved knowledge of family planning [13]. Similarly, in Sierra Leone, the use of mhealth facilitated the dissemination of family planning messages and demand creation [14]. The use of SMS and interactive voice messages have been reported as scalable channels to reach non literate population [13]. Therefore, providing comfortable, at-home education for women and their family members can improve awareness, enable women to make informed choices about their health and well-being and enhance uptake.

Addressing unmet needs for FP requires interventional messages that are designed to target specific groups of women and girls at various intervals and timelines. For example, messages provided during antenatal care visits may differ from messages targeting adolescent girls and women of childbearing age [15–17]. Messages may also differ for women who are pregnant and those who have recently given birth [16]. Moreover, women have reported unique challenges during the postpartum period, including the timing of FP methods, delays in menses after giving birth, and breastfeeding [15]. Designing effective FP intervention messaging requires careful consideration of the target audience and specific cultural and social contexts.

**Fig. 1:**
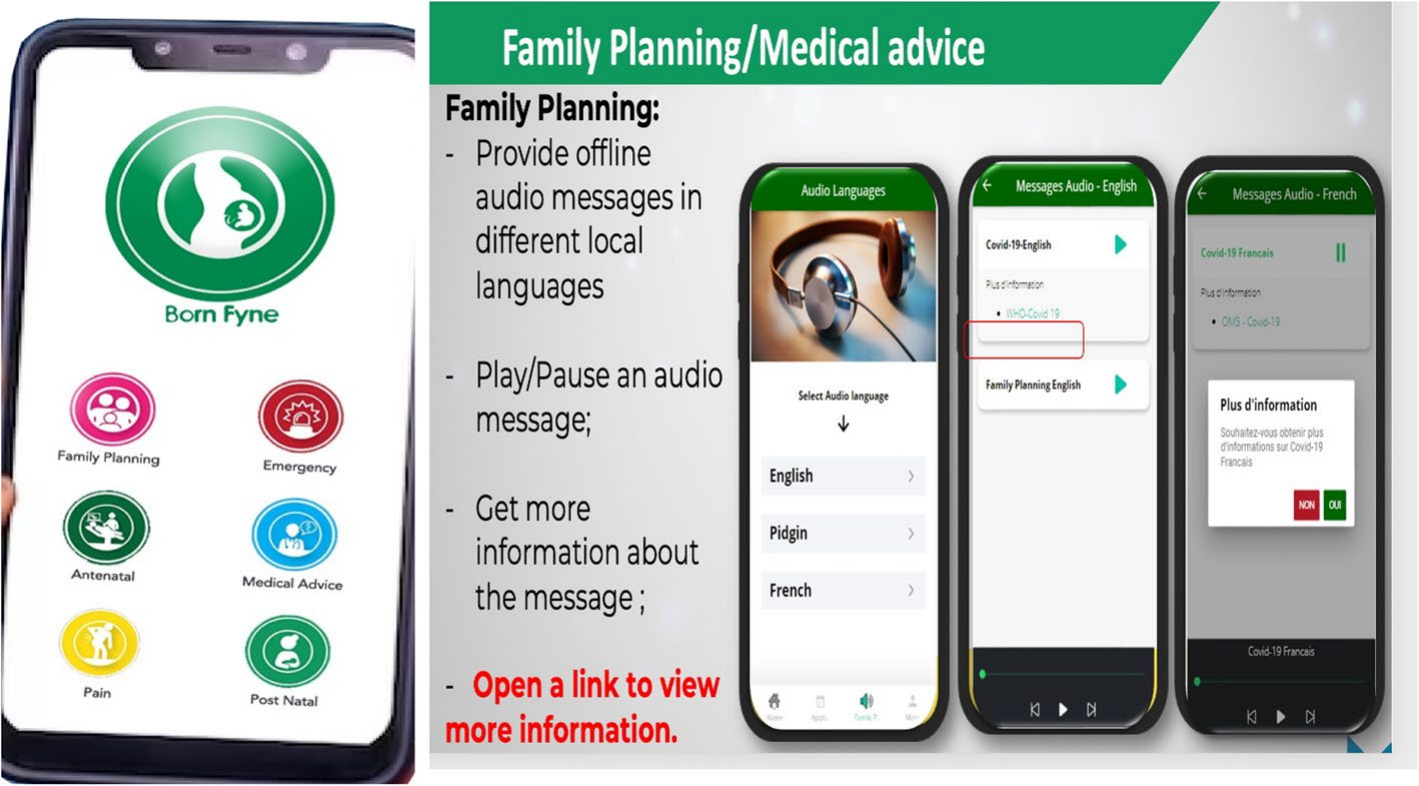
Family planning feature on the BornFyne-PNMS platform

Cameroon recently launched the FP 2030 with three key priorities: i) reducing unmet needs for FP from 23% to 10%%, ii) increasing the use of contraceptives from 15.4% to 35%. and iii) mobilizing the family sector to provide family planning services [8]. In an attempt to address some of the gaps in FP knowledge and unmet needs for contraceptives uptake amongst women in rural setting, the BornFyne-prenatal management system (PNMS), a two-way interactive digital platform with six features including an offline module for family planning (Fig. 1), was developed to support interventional educational messages tailored to specific population group [18–20]. This offline FP module was designed as an educational platform for the continuous education of women by uploading interventional FP messages and content in various local dialects [20]. Providing a continuous educational platform for women on FP services and contraceptives can increase awareness and knowledge and enhance uptake of FP services and contraceptives. The objective of this paper was to document the steps employed in the design and content contextualization of tailored interventional FP messages for the BornFyne-PNMS family planning feature [20] to target specific population in need of FP services.

## Method

### Framework

The study employed the health belief model (HBM) which has been extensively used in multiple health contexts to predict preventive health behaviours and develop interventions [21, 22]. The HBM is comprised of several components that, in combination, can predict behaviour change [21, 22]. We employed this model to explore and understand the gap in unmet needs for FP and to inform the contextualization of tailored intervention messages for the BornFyne-PNMS FP feature. According to the HBM, a perceived threat refers to an individual’s assessment of the likelihood of a negative health condition occurring as a result of a risk or issue [21]. On this basis, the HBM was used to explore and assess participants’ responses to the use of contraceptives and practice of FP in general and to capture their understanding and gaps on contraceptives. Perceived severity refers to an individual’s assessment of the seriousness of contracting a health condition and the potential consequences; this measure was used to understand participants’ perspectives on the use of contraceptives and their knowledge of FP in general, as well as any perceived advantages or disadvantages. The likelihood of an individual committing an action is weighed by comparing the perceived benefits—which are the rewards attributed to engaging in the recommended behaviour—to the perceived barriers or obstacles that prevent the individual from engaging in the recommended behavior [21]. Cues to action relate to motivating factors that may provoke or encourage a change in behaviour, and self-efficacy refers to one’s belief in their ability to perform and maintain the desired behavioural change [21]. This model was used to identify and understand factors that may trigger changes and to obtain a more comprehensive understanding of the community’s perspective on FP. This, in turn, helped guide the design of tailored FP intervention messages to target the specific needs of the community.

### Study setting

Cameroon is a lower-middle-income country with an estimated population of 26 million as of 2020 [11, 22]. Women comprise approximately 50% of the population [11, 23]. Adolescents and young people aged 10 to 24 make up approximately 34% of Cameroon’s population [11]. Healthcare is financed mainly through out-of-pocket expenditure [11, 23]. Cameroon is made up of 10 regions, 8 regions are predominantly French and 2 are predominantly English. The BornFyne-PNMS project is currently implemented in four districts in Cameroon, two from French and two from the English regions, namely, Bangem and Tiko (English, Southwest region) and Akonolinga and Ayos (French, Central region). The districts were purposefully selected to ensure the representation of both French (Ayos and Akonolinga) and English (Tiko and Bangem) regions, and a mix of rural and urban settings as defined in the context. In 2017, the country adopted a performance-based financing program that pays providers incentives based on predefined quality and quantity criteria; this program is a national strategy intended to improve reproductive maternal health services as well as the performance of health providers [24]. In 2023, the country official launched a pilot universal healthcare program which is tested in selected districts [25].

### Study design

This study is a mixed method explanatory sequential design that included a cross-sectional survey of households and a focus group discussion (FGD). This study is part of a larger study for the BornFyne-PNMS digital platform, and this paper describes the process of contextualizing the FP messages for the BornFyne digital platform. We conducted a survey in the four districts to gather households’ perspectives on family planning and contraceptives. Given that BornFyne-PNMS is a digital platform, the survey was to gain a situational assessment of the community’s perspective on listening to family planning messages from their mobile phones and in their local dialect and to capture a snapshot of households with a mobile phone or smartphone in the respective districts. In addition, the survey questions explored network availability and connectivity and to obtain a broader perspective on households’ understanding of FP and the methods and barriers to addressing unmet needs. This survey was followed by a focus group discussion with women and men identified from the surveyed households. In addition, we conducted focus group discussions with community health workers (CHWs) in each district.

### Sampling

**Fig. 2:**
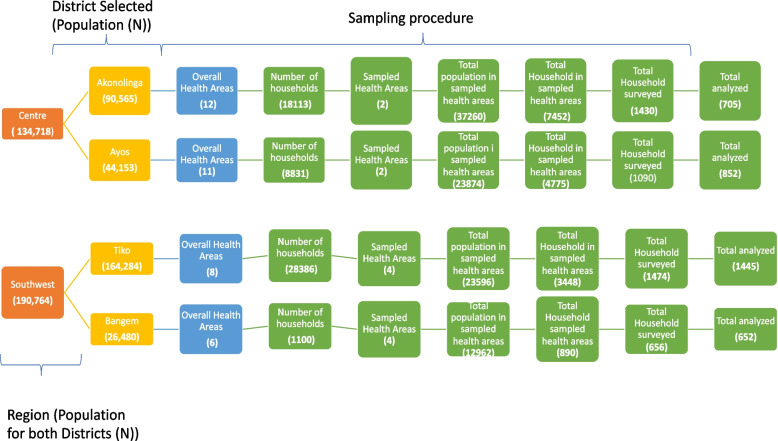
Sampling frame

In order to determine the percentage of households with mobile phones in each district, we planned to survey all households in each of the four districts. However, due to limited resources, we reduced the number of households surveyed. The survey was designed as a door-to-door assessment to be performed by data collectors who are familiar with the target health district, have worked on similar projects, and have conducted home visits in the past. Based on data gathered from the districts, the estimated number of households was 18,113 in Akonolinga, 8,831 in Ayos, 1100 in Bangem, and 28,386 in Tiko (see Fig. 1). Each district is divided into the following health areas: Akonolinga (12), Ayos (11), Bangem (6), and Tiko (8). Due to limited funds, we determined the number of households to be surveyed based on the cost per household visit, the availability of project resources, and the timeline. Given that the health areas for the entire BornFyne study was selected based on a facility assessment tool conducted to assess the quality of all health facilities in the area, as well as distances from intervention and control health areas, the survey was conducted in the selected health areas. The selected health areas were further divided into zones or quarters to facilitate data collection, and each data collector was assigned to a specific zone or quarter within their respective district (see Fig. 2).

Using a proportionate sampling approach, we estimated the total number of households to be surveyed per district based on the estimated cost of 3,000 CFA Francs per day and a maximum of 30 households per data collector per day. Thus, it was anticipated (based on the number of households in the area) that a total of 1,300 households would be surveyed in Akonolinga, 1,000 in Ayos, 2,000 in Tiko, and 800 in Bangem (see Fig. 2).

The survey contained 20 questions that are related to family planning, including demographic questions. Respondents were asked to answer yes or no, and a 3-point Likert scale was employed. The door-to-door survey employed a convenient sample, and any household that refused to participate was skipped. Instead, the next household was visited until the timeline for the survey was reached or resources for the survey were exhausted. The survey lasted for 20 min, and informed consent was administered to all households. The questions were categorized using themes from the HBM, which enabled us to explore perceived benefits in relation to household respondents’ understanding of FP as well as their knowledge and use of contraceptives. For example, the survey asked such questions as “Have you heard of FP before?” Have you or any one in your household used any form of FP before?” And “Can you list any form of FP method, or do you know any FP methods?” The perceived threat and severity category was used to explore participants’ experiences using FP methods and their potential side effects. The participants surveyed were also asked if they could list any advantages of using FP as well as any barriers to access.

### Focus group discussions

Participants were invited to the FGDs based on their responses to family planning questions (especially in relation to side effects, contraceptive use, and post-partum), however, some participants were invited in the focus group discussions based on referrals from CHW. The survey questions were the same questions that were explored in the focus group discussions. The discussion used a semi-structured format that allowed us to generate additional information on participants’ understandings of FP. Each focus group ranged from six to twelve participants for each district, and each group contained women, men, and CHWs. The FGD for CHW was to provide additional insights to the respondents (women and men) perspective of FP and to guide and inform strategies for the contextualization process. The FGDs lasted for 1.5 h, and no relationship was established with the participants before the FGDs began. The FGDs were conducted using pidgin English or French, depending on the dominant language of the district, and it was coordinated by MN, FW, VN, and OK, all of whom are familiar with both languages. The sociodemographic characteristics of respondents were collected at the end of the discussion. Participants were allowed to choose a convenient location for holding the focus group discussion, and participants were provided transportation at the end of the discussion.

### Data analysis

The survey was analyzed using statistical analysis software to generate descriptive statistics based on participants’ views and are presented using the subcategories from the HBM. Incomplete responses from the survey were removed from the analysis.

The content of the FGDs from the French respondents was translated into English and transcribed by research assistants (RS and EK) whom are both bilingual and all familiar with the languages. The translated version was later reviewed by the team for accuracy and validation. Data was exported into MaxQDA for analysis. Data analysis of the focus group discussions was done using thematic analysis, incorporating the themes from the HBM. Data analysis was performed by two independent coders, MN and MNN. The coded elements were reviewed and compared by AF and PO for agreement. Responses were further grouped based on each category, and elements that explained the knowledge gaps and areas in which participants required additional information were considered important for further education see Table 1. These items were grouped, and member checked with selected participants from each district who were part of the FGDs.

**Table 1:** Using the Health Belief Model to identify gaps and unmet needs to inform family planning messages

Themes	Categories	Subcategories	Excerpt	Knowledge gaps/unmet needs
Perceived benefits	Positive experiences covering social and economic latent implications	Preventing nutritional deficiencies and health issues	*“You do family planning so that they can achieve their goals by giving birth to the number or children they want. They also so it to protect the children so they don’t get malnutrition, lack motherly love. When you are pregnant back-to-back (get belle on top belle) it causes the current child not to grow well. They do not suck breast long enough to get the vital nutrients. The food you are supposed to give the young child is not sufficient and the one you are pregnant with is your main concern. You are mostly concentrated on the one you are pregnant with. The one in your arm begins to look malnourished and abandoned”*. [FGD Tiko women].	
Perceived barriers	Lack of awareness, limited information knowledge	Communication, unavailability of female condoms	“*To have a discussion is not easy even to reach to the level of her showing you her cycle on a calendar. She can even tell you that if you master her cycle, you have mastered her, so it very difficult. Even if it’s not easy, there are some women that say they’ve mastered their cycle, don’t fall into the trap in the end”*. [FGD Akonolinga men] *“In family planning, we laid emphasis on male condom, so when the female condom was out, it did not go loud as male condoms. So that is why you will see there are some men who have never seen it with their eyes because there was more interest on male condom. That what made it not to be known. If you walk in stores, when you want to buy a condom, you cannot find a female condom. It is mostly male condom”[FGD Tiko Men].*	Poor communication, limited education, sensitization efforts on male versus female condoms
Perceived susceptibility	Vulnerability of men and women, young and adolescent girls and boys	Negligence from women, weakness from men, cost, financial status, quality	*“If I don’t have money to take care of the baby, I tell her that she is disturbing. A child is not a toy. A child is a responsibility. I am saying this. If my wife says that she doesn’t want condom and want it direct, if I don’t have money to handle a child how will I do?”* *“just wish to add something, when you go to a shop to buy a condom, the prices tells you the type of condom you’re buying. When you go and they’re telling you this condom is 100cfa and a different one is 150cfa and another 200cfa. At times we prefer to buy the one for 100cfa. Just that price, should tell you the type of condom, because when you decide to go to the shop for 200cfa. For example, a condom costs 200cfa. Those who are not financially capable, will go for the one for 100cfa”*.[FGD Men Tiko/Akonolinga]	Potential cost implications,
Perceived severity	Negative experiences of contraceptive use	Weight gain, loss weight, bleeding,	“*I’m afraid if it because when they place it, some people bleed a lot (bleed sotey), some people gain weight. I’m fat, so I don’t want something that will make me so fat that I can’t fit through the door. There’s another that makes you lose weight, lose appetite (no di chop fine) I don’t want any of such”.[*FGD Women Bangem/Tiko] *A woman that was teaching walked pass my door and told me about family planning. She told me that when I removed the family planning, I was still very fertile and wasn’t supposed to sleep with my partner. I didn’t know and the person that removed it also didn’t tell me anything. Once I removed it, in less than a month I got pregnant. (FGD women Tiko)* *After giving birth I went for them to place it. It really disturbed me and in less than 2 months I went back for them to remove it. As I removed it, in less than a month I got pregnant”*.*”*.( (FGD Women Tiko] *The woman that was giving it to me didn’t explain it to me. She just said it’s good as it prevents me from getting pregnant.For the whole month I used it, it really disturbed me. I was menstruating too much. As the 1 month came to an end, for my next appointment I decided to stop it”*.( (FGD Women Tiko]	Limited knowledge and education on side effects, post-partum timing
Self-efficacy			“*You could have a stable partner, but he strays (il pars dehors) you can still use it, it’s not only to avoid pregnancy. There’re men in the townhall that are married, and they stray, the women can be the faithful. When he comes back you don’t know what he has done outside so use the condom, thats how you’ll avoid illnesses. I don’t joke with that. I have a husband, but I use it”.[* Women Akonolonga FGD]	
Cues to action	Listening to family planning messages frequently, dialogue and consensus	Motivating factors, local language, dialogue, consensus, education, Training, male engagement	*“Yes, it’ll be the best. In English there’ll use big terms which will confuse us further. And if we need to ask questions about that term at that time, we may not be able to reach you. It’s better to use a local language that we know”*. [FGD Women Akonolinga] *“Because those young ones outside, if they have such information on an application, which helps educate them, she will talk of it to a friend and at the end they will understand the necessity of this application. The beginning will be difficult. When you don’t understand you fell like it is difficult. But it is an innovation. Because you will talk with a doctor, me I talk to a doctor who treated me, and I have never seen him with my eyes. I explained my problem and he told me what to do and finally I was treated. Especially when people want”*. [FGD men Akonolinga/Tiko]	Language used in communicating family planning Limited education targeted to youths and adolescent,

The listed points in Table 2 (informed by Table 1) were further discussed with the representative of the Ministry of Public Health within the Department of Family Health responsible for FP. These elements identified knowledge gaps and unmet needs, and therefore, messages were aimed at addressing these gaps. The representative shared a list of FP messages initially prepared by their team. In addition, a list of messages was gathered from the United Nations Population Fund and United Nations International Children’s Emergency Fund websites [26–37] (https://www.doctissimo.fr/html/sante/mag_2001/mag0427/sa_3940_nexplanon.htm#comment-se-passe-le-retrait-de-l-implant) (see Table 2). Finally, messages targeting postpartum women were also obtained from various sources [26–37] (https://www.doctissimo.fr/html/sante/mag_2001/mag0427/sa_3940_nexplanon.htm#comment-se-passe-le-retrait-de-l-implant). These identified messages, which have been used in different contexts, were further grouped to align with the knowledge gaps identified in Table 1 and presented in Table 2. We adapted and aligned the messages to each item identified as a knowledge gap (see Table 2) to ensure the messages align to the knowledge gap. These messages were finally reviewed and validated by the Department of Family Health and the district team.

**Table 2:** Identifying the intervention messages for family planning for the BornFyne PNMS v2.0

Categories of knowledge gaps for intervention	Description	Excerpts from participants to validate the gap	Sample Intervention messages from UNFPA, UNICEF, WHO, and other sources
**1. Timing of use of family planning, removal, and when to get pregnant**	Participants’ discussion revealed that they knew about family planning, but some areas were of concern to them especially when to start using family planning and after removing the family planning when to get pregnant or how soon they can get pregnant. Most were ignorant of how each method works and limited knowledge and understanding of some of the methods.	*“I was scared that it could happen and that I could no longer give birth. I practically bled for 2 weeks like a woman had just given birth. My mother kept talking because I gave birth at 16yrs. The first injection normally disturbs, but we never know about the 2nd one. I took the 2nd one and after 3 months, I was dry for almost 1 year/ I stopped menstruating for almost one year I had transformed. After 2 years, I had sex, that’s how pregnancy came along”* [FGD Women Akonolinga.] *A woman who was teaching walked past my door and told me about family planning. She told me that when I removed the family planning, I was still very fertile and wasn’t supposed to sleep with my partner. I didn’t know and the person that removed it also didn’t tell me anything. Once I removed it, in less than a month I got pregnant. That’s all about the family planning (FGD women Tiko)*	The implant can be removed at any time (maximum 3 years after placement). On the day of removal, a new implant can be placed in the same place [1]. Ideally, young women and men should not have children before the age of 18, or before they have completed their studies and are ready to do so [1].
**2. Post family planning removal what to do?**	It was understood during the discussion that women were ignorant of how some of the methods should be administered and what to do after removal.	*They told me, that if I wanted to get pregnant, I needed some antibiotics to clean my system. A nurse approached me and advised me to take antibiotics to ‘clean’ my system before I got pregnant, and if I didn’t want to take antibiotics, I should wait for the family planning to finish my system, so that way I could get pregnant. I waited for it to finish in my system until I got pregnant*. (Tiko) For 5 years it *disturbed me a lot. I was pale, wasn’t eating, I looked white as though I didn’t have blood. They advised me to remove it because it was disturbing me* (FGD Women Tiko).	You can become pregnant as early as one week after taking an implant. After implant is withdrawn, and if you do not wish to become pregnant at this time, you must immediately start using another method of contraception, such as condoms. Women may experience mild discomfort and some bruising after implant removal [2]. There are no medical problems caused by a delay in the removal of long-acting methods such as implants or IUDs. Do not attempt to remove the contraceptive method yourself; wait until you have access to health care from a qualified provider [3].
**3. What type of family planning method is best?**	Generally, both men and women across all districts were interested in knowing what type of family planning is best and how to go about it. They were very eager to get answers to specific questions on specific family planning methods, some saw condoms as the best method especially as it also could prevent STIs but at the same they did not understand why a condom should burst Some women saw the natural method as ideal.	*we just go and get it done when we haven’t done blood tests to know if it is compatible with us. I’m speaking for myself, If I want to use it once I’ve given birth, I need to go get checked at the hospital first to know which I will be compatible with before I can take it.* *I’m interested, particularly on the part of choice, since there are many side effects there may be a method that suits your wife. It seems many women have that issue so you may need another method but which? (Akonolinga)*	All modern methods of contraception help prevent pregnancy. Women and their partners can choose any modern contraceptive method that is acceptable and safe for them. There is a wide variety of modern methods, one of which may suit you best. Condoms, when used consistently and correctly, are the only method of birth control that helps prevent unwanted pregnancies and protect against sexually transmitted infections, including HIV. They can be used with other birth control methods to protect against both unwanted pregnancy and sexually transmitted infections. Emergency contraceptive pills can prevent up to 95% of pregnancies when taken within five days of having sex, and they can be taken by anyone with or without a medical condition [3].
**4. Steps to considered before using family planning**		*It is only later that a woman explained family planning to me. She said they usually check the person before knowing which one is good for them. The one for 1 month may have not been good for me, but I could try the one for 3 months or 5 years. Family planning is good, just that it wasn’t good for me, that’s why I stopped*. [ Women FDG Tiko]	The adoption of a contraceptive method may depend on several factors: age, education, physical health, parity, profession, intervention of spouse or partner, rumour, quality of information received on the method [4].
**5. Potential side effects**		*Concerning infertility, when I had the first plan A in implanting, we took a break, and then we went on. When we went on we had my woman conceive. However, she had a stillbirth during delivery, and that’s when we started asking questions. Since I saw so many advantages, I had to keep using it, I’m still wondering whether I should go on after taking on this one or I should carry on with implanting. I’m wondering whether that stillbirth was caused by that family planning or something else, as the diagnosis did not show the cause of it* [Tiko men FGD]	• it can wake up acne problems, • it can promote weight gain in certain predisposed women, • it can lead to menstrual disorders (bleeding between periods, irregular periods, absence of periods) [5],
**6. Who to talk to and when?**	Most respondents expressed challenges in communication especially when and who to talk to. Some men also expressed challenges in communicating with the women. Some women also find it challenging to communicate with men and sometimes engage in family planning practice without informing the husband or partner. When they start experiencing side effects, they will want to involve the husband so some men didn’t see it as the right approach because they believed family planning should be discussed as a family and not only the woman.	*Other methods can change your menstrual cycle in a way that disturbs you a lot and causes you to spend a lot of money. If you meet a doctor that is not experienced, he may insist you took some tablets, when you didn’t take them.* *It’s also difficult for that type of woman to talk about what they’re experiencing if you as a man don’t ask her what is bothering her.*	If you notice any other type of side effect not mentioned above, you should either contact a qualified health care professional, or go to the nearest health facility[ref].
**7. Silo decision making**	Some men are particular about the way some women make decisions without consulting their husbands and they believe this is not right when there is a negative outcome that is when the women will be aware of what they have engaged in and sometimes they have to tendency to blame it on cultural aspects.	*However, in today’s world, many women are making the discussion to undertake family planning by themselves. When a problem arises, they blame it on an elder (ancestors) in the family saying that they are the ones that have blocked them. Though she is the one that has implanted or injected family planning at a young age. (FGD men Tiko)*	Women should talk with their husbands and the husband talk with their women to discuss about family planning for their household
**8. Family and household education and communication**		*I will add in the same light by saying that it is in our customs in Africa generally when 2 people get together, it should automatically result in making children. There is this education that Africans receive, a woman who does not have children in her home is not considered a woman. On top of that, we Africans have received an education that a child represents wealth, so the one who has more children has an abundant workforce that can allow him to become someone well-positioned in life, which makes it difficult to ask Africans to go to a family planning because it is in the education. This vision will come with time because it is necessary to advise people a lot, and it is necessary to raise awareness. I think that awareness will come much more from the man’s side because it is the woman who carries the child, it is the woman who undergoes everything, but the man is the one who puts the child, he only waits for the day and this is why in couples it is necessary to raise awareness among men. Because the real difficulty is at the level of men, women do it without telling their husbands and the consequence is that many of them do not manage to conceive in a short time. This means that when they stop using this method to conceive again, it becomes difficult* [FGD Men Akonolinga/Tiko]	Family planning allow young girls and boys, women, men and couples to determine the number and timing of pregnancies through the use of a method of contraception.
**9. Youths and adolescent girls and boys**		*Nowadays, what we notice is that most youngsters don’t use condoms. Maybe the boy will want to use the condom but the girl will refuse saying that the condom reduces the pleasure. So, when the girl says that, you the guy must obey because you want to satisfy your partner. They usually say “We don’t eat bananas with its skin”. So, to eat a banana you must remove the skin. If you insist, she will refuse. And that is how we go on for it.* *Parents should really emphasize the education of their children, especially young girls. They are vulnerable. We should prompt parents to discuss with their children, boy or girl, we don’t know where the problem can come from*	For the girl, from the onset of menstruation. Parental consent is mandatory. But ideally from 15 years old
**10. Post-partum period for women after given birth**		*After giving birth I went for them to place it. It really disturbed me and in less than 2 months I went back for them to remove it. As I removed it, in less than a month I got pregnant. (The woman that was giving it to me didn’t explain it to me. She just said it’s good as it prevents me from getting pregnant. (Tiko)*	For the woman who has just given birth, it is advisable, immediately after childbirth, to use a method of family planning to avoid pregnancy. If you do not practice exclusive breastfeeding immediately after delivery, it is advisable to use another form of contraception.

**Fig. 3:**
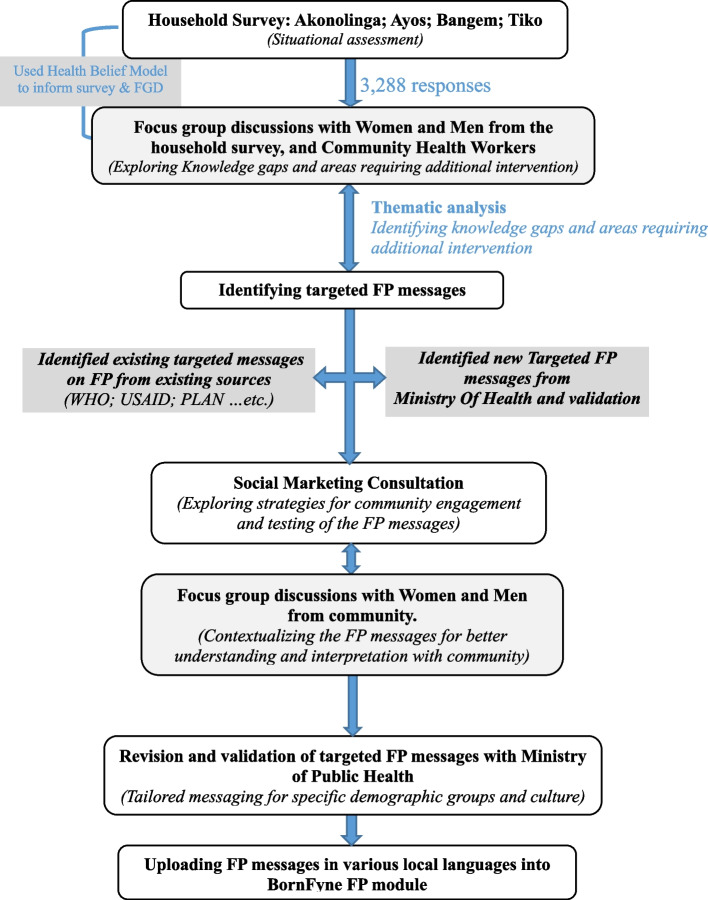
Overview of the steps in contextualizing the family planning messages for the BornFyne-PNMS

Next, the validated messages were reviewed by a team of experts from a social marketing organization in Cameroon to ensure that the messaging content would be easily understandable by community members and to obtain expert feedback (see Fig. 3 on the steps). As a result, the research team, together with the social marketing team designed an evaluation scale with four items to assess the community’s understanding of the messages, as well as to determine whether there was a need for more information and whether the wordings, meanings, and interpretations were easily understandable. The research team, together with the social marketing team, tested the messages using the following methodology, which was also informed by the social marketing team. A total of 50 messages were tested with a group of literate and non-literate men and women from each district. A total of 80 participants (20 per district) who were not part of the FGDs from each district were used to test the messages, review the content and provide feedback, including five literate men and five non-literate men, as well as five literate women and five non-literate women of different age groups per district. A Likert scale was used to assess their level of comfort in understanding the messages (1: I don’t understand; 2: I partially understand; 3: I understand; and 4: I completely understand). The outcome was focused on the number of messages that were considered difficult to understand (i.e., “I don’t understand”) or completely understood (see Table 3). This was done for each district. When participants identified certain messages or wordings as needing revision, they were asked to provide a synonym or suggestion on how the message or wording could be changed to enhance understanding among members of their household and/or community. Members were asked to respond if they understand and to raise concerns if there is any word that they understood but think it would be difficult for members of the household or community to understand. The messages were refined using excerpts from participants’ statements; for example, participants suggested the word “menstruation” in a sentence to be changed to “period” to reflect contextual meaning and definitions. The final messages were uploaded by the district health team to the BornFyne-PNMS FP system in four languages: French, English, pidgin English, and other local dialects recommended by the respondents. They were also presented at the launch of the Family Planning 2030 ceremony in Cameroon [8].

**Table 3:** Results of the messages tested with participants across all four districts

	**Number of messages deemed difficult or impossible to understand**		**Number of messages deemed well or perfectly understandable**	
	Number of messages (*N* = 50)	%	Number of messages (*N* = 50)	%
Women unable to read and write	24	48%	26	52%
Women who can read and write	22	44%	28	56%
Men unable to read or write	23	46%	27	54%
Men who can read or write	21	42%	29	80%

### Ethics approval

Ethical approval was obtained from the National Ethics Board of Cameroon ref # 2022/07/1467/CE/CNERSH/SP and the University of Ottawa Social Science Ethics board ref #H-05-22-8077. Administrative clearances were obtained from the Ministry of Public Health at the national level in Cameroon (ref. D30-1440 No. 631–3822) in collaboration with the Division for Health Operations Research (DROS) in Cameroon, the southwest regional delegation of public health (ref. P412/MINSANTE/SWR/RDPH/CB:PF/941/618), and the Central Regional Delegation of Public Health (ref. 1393–4/AAR/MINSANTE/SG/DRSPC).

## Results

### Quantitative results

A total of 705 responses were analyzed from Akonolinga, 885 from Ayos, 1,747 from Tiko, and 656 from Bangem (see Table 4). Female respondents were surveyed more in Tiko and Bangem, and mostly male respondents in Akonolinga. Ayos represented a more balanced gender response rate for men and women. Most of the respondents across the four districts were married followed by single respondents with a mean age range of 34–42 (Table 5). The highest education level of the majority of the respondents across the districts was secondary school. Most household respondents across the four districts reported farming as the main activity, and more respondents from Tiko reported to be self-employed. Most reported religious status was Catholic followed by Presbyterian.

**Table 4:** Socio-demographic characteristics of respondents for the household survey

Demographic Characteristics	Tiko (*N* = 1445)	Ayos (*N* = 852)	Akonolinga (*N* = 705)	Bangem (*N* = 652)
	**n (%)**	**n (%)**	**n (%)**	**n (%)**
**Gender**				
Males	362(25.1)	404(47.4)	436(61.8)	132(20.2)
Females	1083(74.9)	448(52.6)	269(38.2)	520(79.8)
**Marital Status**				
Married	778(53.8)	257(30.2)	186(26.4)	433(66.4)
Divorced	38(2.6)	8(0.9)	13(1.8)	11(1.7)
Single	443(30.7)	381(44.7)	404(57.3)	159(24.4)
Widowed	160(11.1)	46(5.4)	68(9.6)	42(6.4)
Prefer not to say	26(1.8)	160(18.8)	34(4.8)	7(1.1)
**Education**				
Primary	522(36.1)	137(16.1)	124(17.6)	179(27.5)
Secondary	580(40.1)	359(42.1)	297(42.1)	242(37.1)
High School	259(17.9)	152(17.8)	191(27.1)	127(19.5)
Bachelors	33(2.3)	97(11.4)	35(5.0)	75(11.5)
Masters	6(0.4)	4(0.5)	2(0.3)	3(0.5)
PhD	5(0.3)	9(1.1)	8(1.1)	0(0.0)
No formal education	40(2.8)	27(3.2)	2(0.3)	18(2.8)
Other	0)0.0)	67(7.9)	46(6.5)	8(1.2)
**Religion**				
Catholic	447(31%)	409 (40%)	117 (17%)	333(51%)
Presbyterian	499(34%)	367 (36%)	119 (17%)	160(25%)
Pentecostal	364(25%)	58 (4%)	21 (2%)	95(14%)
Muslim	28 (2%)	52 (5%)	5 (1%)	4(1%)
No religion	22 (2%)	11 (1%)	4 (1%)	11(2%)
Other	103(7%)	116 (11%)	4 (1%)	45 (7%)
**Employment**				
Employed for salary	81(5.8%)	75 (8.8%)	77 (11.22%)	54(10.2%)
Student	84 (6%)	65(7.6%)	13 (1.8%)	72 (13.6%)
Farming	214(15.3%)	300 (35.2%)	305 (44%)	178 (33.7%)
Business (self-employed)	663 (47.6%)	175 (20.5%)	95 (14%)	26 (4.9%)
Housewife	201(14.4)	106(12.4%)	23 (3%)	126 (23.8%)
Retired	57 (4%)	21(2.5%)	23 (3%)	8 (1.5%)
Not working	90 (6.4%)	108 (12.7%)	150 (22%)	64 (12.12%)
**Language most spoken at home**				
English	138(9.5%)	4(0%)	1 (0%)	183(29%)
French	28 (2%)	354 (37%)	120 (44%)	5(1%)
Pidgin English	1270(88%)	3 (0%)	1 (0%)	65(10%)
Dialect	9(0.6%)	579 (60%)	358(52%)	384(60%)
Other	0(0%)	23(0%)	1 (0%)	2(0%)

**Table 5:** Mean age and characteristics of household respondents

	**Tiko ** ***N*** ** = 1474**		**Bangem ** ***N*** ** = 656**		**Ayos ** ***N*** ** = 885**		**Akonolinga ** ***N*** ** = 705**	
	**Mean**	**SD**	**Mean**	**SD**	**Mean**	**SD**	**Mean**	**SD**
**Age of survey respondents**	41.27	13.56	34.88	11.58	36.46	12.23	42.67	15.11
**How many people live in your household in total?**	5.41	2.68	5.99	2.69	6.16	2.53	6.69	3.35
**Number of males in your household**	2.32	1.62	2.85	2.05	5.20	2.87	4.38	3.33
**Number of females in your household**	3.15	1.85	3.23	1.68	2.77	1.65	2.69	2.05
**Total adolescent per household**	1.43	1.52	1.59	1.54	2.86	2.02	2.85	1.96

### Socio-demographic characteristics of respondents

#### Using the health belief model to understand family planning knowledge, gaps and unmet needs

##### Survey results

Employing the Health Belief Model, where we aimed to comprehend knowledge gaps and unmet needs regarding family planning among participants. The household survey results revealed intriguing insights into participants’ perceptions, experiences, and preferences related to family planning.

##### Perceived benefit and knowledge

**Fig. 4:**
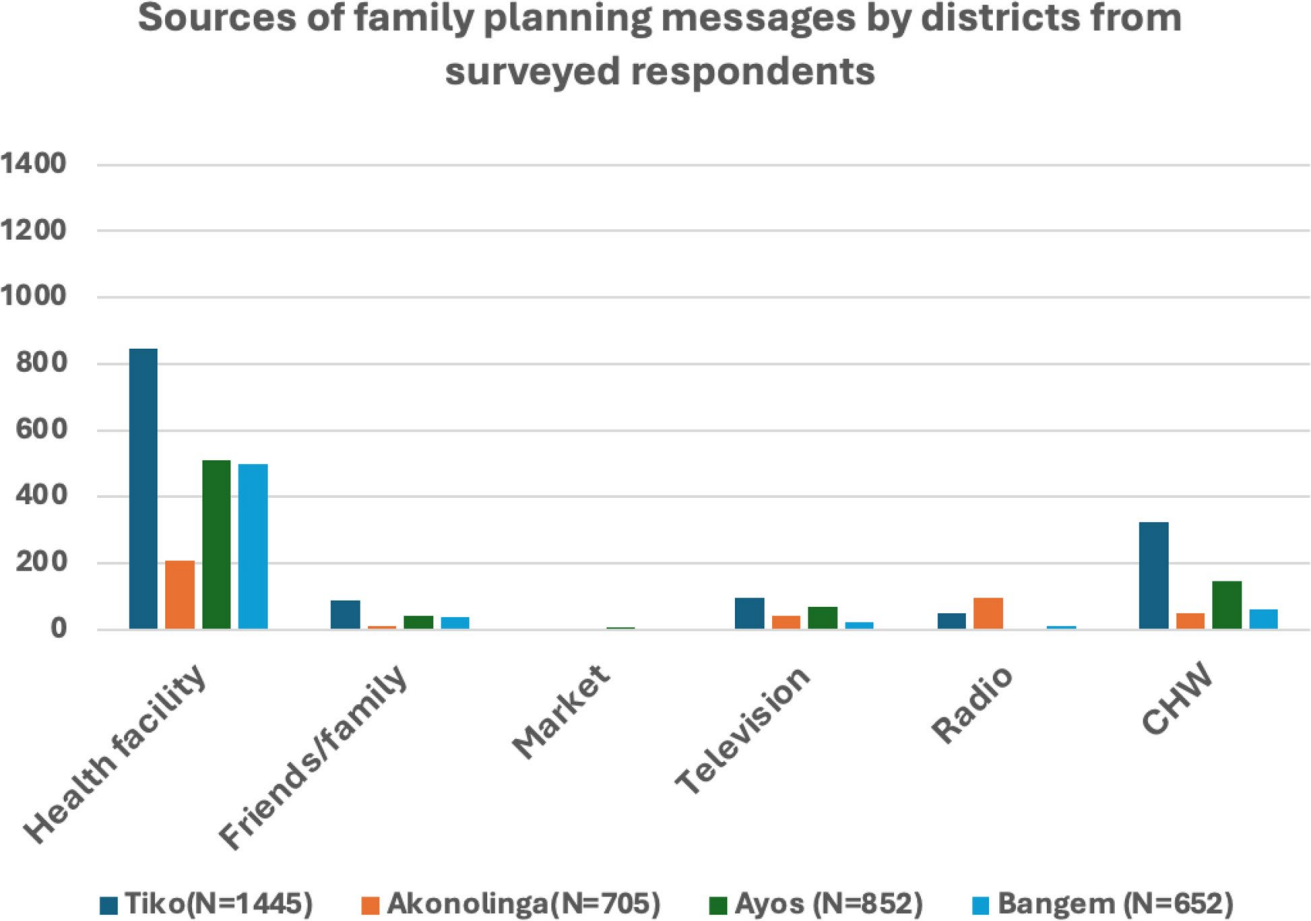
Number of households that responded on their sources of family planning information

A majority of participants in the survey from each district (over 80%) acknowledged the benefits of family planning, with a considerable portion of women having utilized family planning methods before (over 60%) (Table 6). Notably, respondents from all districts primarily received family planning messages from health facilities, followed by community health workers in Tiko, Ayos, and Bangem. Akonolinga stood out, reporting radio and television as the second most common sources (see Fig. 4). Overall, 95% of household respondents had prior knowledge of family planning, underscoring the prevalence of awareness across the study areas.

##### Media preferences for family planning messages

Media preferences varied across districts, with over 80% of respondents in Ayos and Bangem expressing a willingness to receive family planning messages via mobile phones. In contrast, Tiko and Akonolinga exhibited different patterns, with 55% and 15%, respectively, showing interest in this mode. Understanding these preferences is crucial for tailoring communication strategies to effectively reach diverse communities see Table 6 below.

**Table 6:** Perceptions and gaps in family planning based on responses from the household survey

Questions	Response	Tiko *N* = 1474	Akonolinga *N* = 705	Ayos *N* = 885	Bangem *N* = (656)
		**n(%)**	**n(%)**	**n(%)**	**n(%)**
**Heard of Family Planning before?**	**Yes**	1406(95,39)	367(52,06)	842(95,14)	643(98,02)
	**No**	26(1,76)	41(5,82)	38(4,29)	7(1,07)
	**No response**	42(2,85)	297(42,13)	5(0,56)	6(0,91)
**Used Family Planning Methods before?**	**Yes**	909(61,67)	180(25,53)	650(73,45)	574(87,5)
	**No**	456(30,94)	225(31,91)	203(22,94)	70(10,67)
	**No response**	109(7,39)	300(42,55)	32(3,62)	12(1,83)
**Willingness to receive Family Planning Messages on mobile phone**	**Yes**	823(55,83)	106(15,04)	774(87,46)	534(81,4)
	**No**	607(41,18)	307(43,55)	94(10,62)	107(16,31)
	**No response**	44(2,99)	292(41,42)	17(1,92)	15(2,29)
**Perception on necessity of Family Planning**	**Yes**	1254(85,07)	396(56,17)	857(96,84)	632(96,34)
	**No**	140(9,5)	7(0,99)	16(1,81)	15(2,29)
	**No response**	80(5,43)	302(42,84)	12(1,36)	9(1,37)
**Recommendation on use of Family Planning methods to family members or Friends**	**Yes**	1112(75,44)	197(27,94)	811(91,64)	587(89,48)
	**No**	312(21,17)	211(29,93)	61(6,89)	56(8,54)
	**No response**	50(3,39)	297(42,13)	13(1,47)	13(1,98)
**Experience any side effects using family planning? (only asked to respondents who said yes, they’ve used family planning before)**	**Yes**	92(10)	30(17)	62(10)	32(6%)
	**No**	817(90)	150(83)	588(90)	542(94%)

##### Perceived severity

Some participants reported experiencing some side effects and this was more in Akonolinga (see Table 6 above). These side effects were further explored in the FGD and discussed in detailed in the subsequent section.

##### Cues to action

**Fig. 5:**
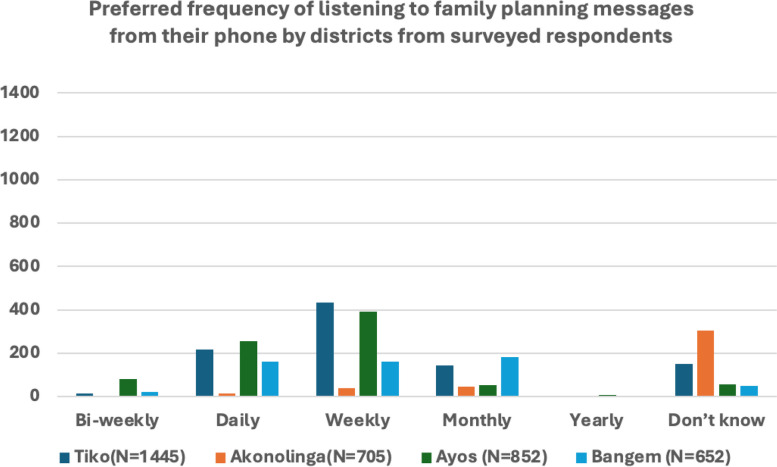
Household responses based on frequency of listening to family messages from a mobile phone distributed over time

**Fig. 6:**
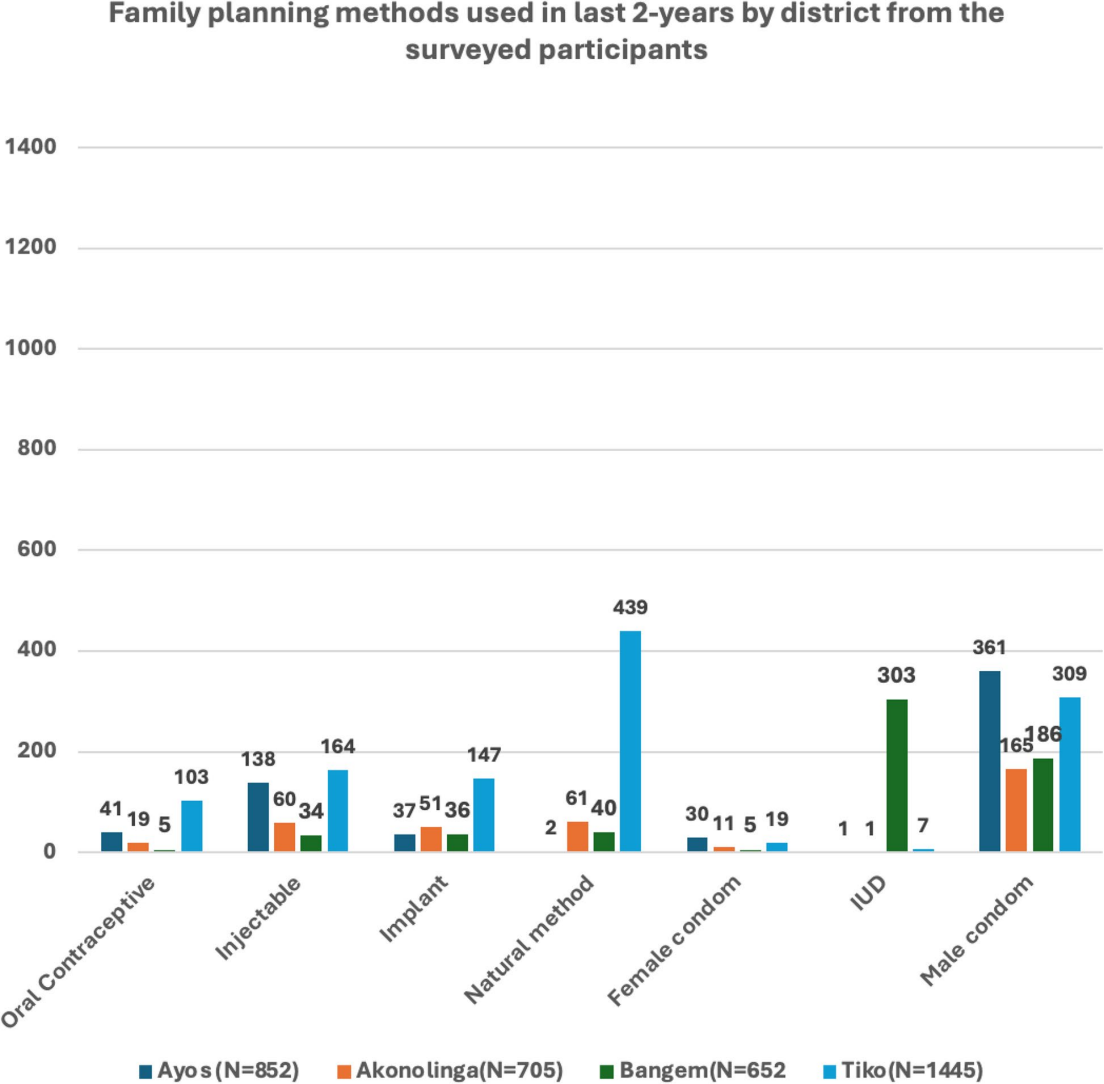
Number of household responses on preferred language for family planning

Majority of participants expressed a preference for receiving family planning messages on a weekly basis (Fig. 5). Tiko emerged as the district with the highest demand for weekly messages, underscoring the desire for consistent and regular information dissemination. Following Tiko, Ayos, Bangem, and Akonolinga showed decreasing levels of preference for weekly messages. The desire for daily family planning messages was also evident, albeit with a lower frequency compared to weekly preferences. While most preferred more frequent updates, a notable proportion expressed a preference for monthly FP messages. Also, respondents’ preference on language used for communicating FP messages varied according to the language that is most spoken in the district and/or household (Fig. 6). This suggests the need for a diverse messaging schedule to cater to varying preferences and the language use in communicating the message. Very few participants indicated a preference for yearly messages. Additionally, a substantial number did not specify any particular frequency, suggesting an openness to various communication schedules. The diverse preferences observed across districts emphasize the importance of flexibility in communication strategies. While a majority favors weekly messages, acknowledging the demand for daily, monthly, and less frequent updates is crucial. Tailoring family planning messages to meet these varied preferences ensures a more inclusive and effective outreach. Recognizing the district-specific nuances revealed in this study provides valuable insights for crafting nuanced communication plans that resonate with the diverse preferences of participants in Cameroon.

**Fig. 7:**
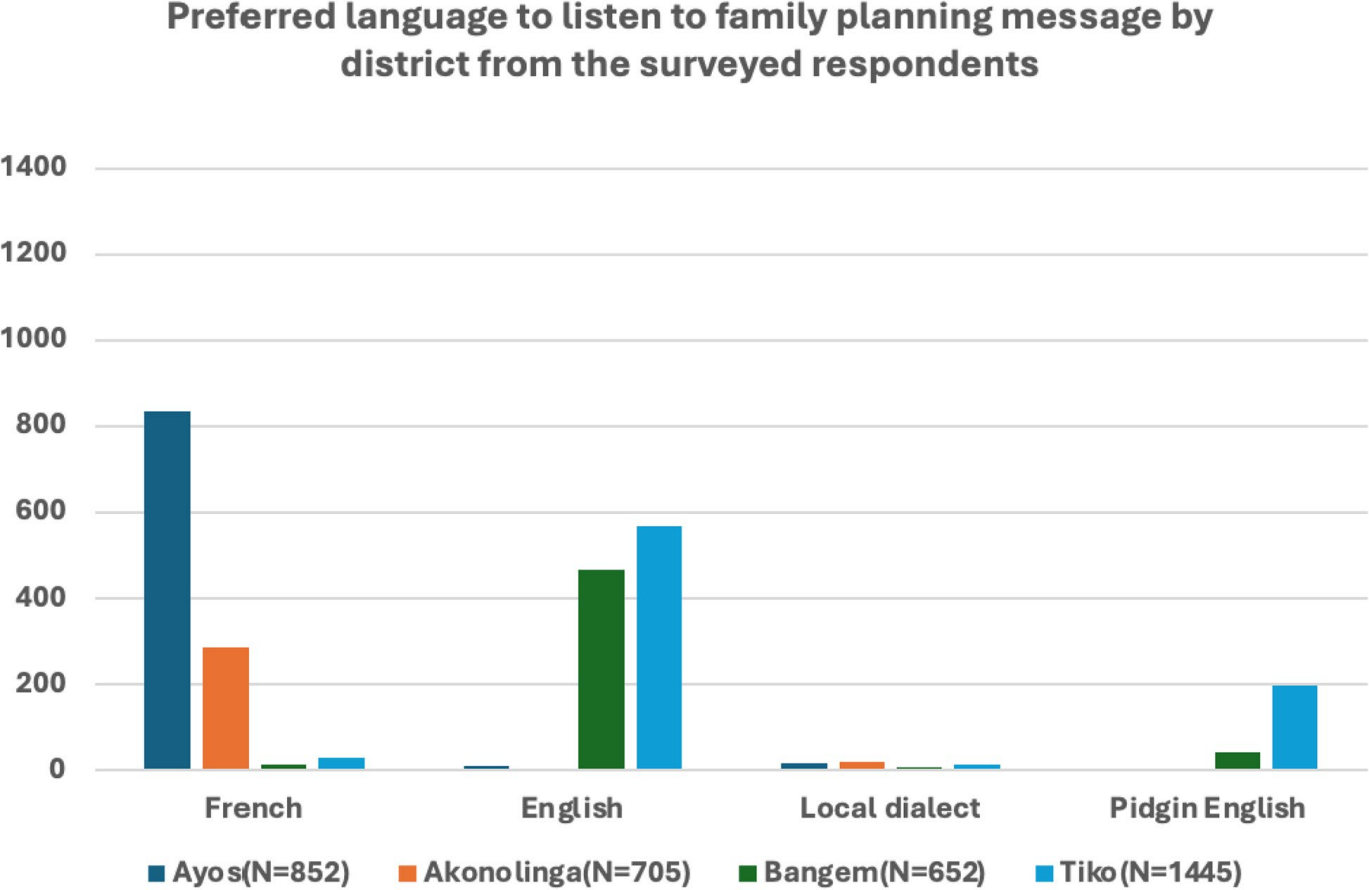
Number of household responses on the method of family planning used in the last 2-years

Participants listed a variety of methods that they have used with male condoms being the most used in the last two years followed by implant and injectable (see Fig. 7). Interesting the natural method was reported more in Tiko.

##### Results of the focus group discussion

A total of 12 FGDs were organized, three from each district. One for women, one for men, and one for CHW. Each FGD composed of 6 to maximum of 12 respondents (see Table 7). The responses are grouped according to themes from health belief model as described below with identification of the gaps and areas of intervention based on participants responses as illustrated in Table 7. The results below present the patterns that emerged from participants responses in line with categories from the HBM and informed the process of identifying the gaps (also presented in Table 1) and identification of the interventional messages.

**Table 7:** Individual distribution of respondents for FGD by district including demographics characteristics

Participants	District	Age	Gender	Occupation	Education
Men	Ayos	29	Male	Student Nurse	Student
Men	Ayos	29	Male	Electrician	Secondary
Men	Ayos	24	Male	-	Secondary
Men	Ayos	36	Male	-	High School
Men	Akonolinga	20	Male	-	High School
Men	Akonolinga	44	Male	-	Secondary
Men	Akonolinga	44	Male	-	Secondary
Men	Akonolinga	42	Male	-	Secondary
Men	Akonolinga	20	Male	-	High School
Men	Akonolinga	21	Male	-	High School
Men	Akonolinga	50	Male	-	Primary
Men	Akonolinga	22	Male	-	High School
Men	Tiko	42	Male	Farmer	
Men	Tiko	37	Male	Teacher	Secondary
Men	Tiko	55	Male	Farmer	-
Men	Tiko	38	Male	Trader	-
Men	Tiko	36	Male	Trader	-
Men	Tiko	30	Male	Mechanic	-
Men	Tiko	34	Male	Driver	-
Men	Tiko	28	Male	Teacher	-
Men	Bangem	39	Male	Farmer	-
Men	Bangem	32	Male	Farmer	-
Men	Bangem	48	Male	Trader	-
Men	Bangem	37	Male	Farmer	-
Men	Bangem	50	Male	Farmer	-
Pregnant Women (PW)	Ayos	18	Female	Student	Secondary
Pregnant Women (PW)	Ayos	23	Female	Student	University
Pregnant Women (PW)	Ayos	32	Female	Nurse	High School Diploma
Pregnant Women (PW)	Ayos	22	Female	Student	University
Pregnant Women (PW)	Ayos	24	Female	Student	University
Pregnant Women (PW)	Akonolinga	35	Female	-	Secondary
Pregnant Women (PW)	Akonolinga	30	Female	-	Primary
Pregnant Women (PW)	Akonolinga	33	Female	-	Primary
Pregnant Women (PW)	Akonolinga	16	Female	-	Secondary
Pregnant Women (PW)	Akonolinga	15	Female	-	Primary
Pregnant Women (PW)	Akonolinga	16	Female	-	Primary
Pregnant Women (PW)	Akonolinga	30	Female	Student	University
Pregnant Women (PW)	Akonolinga	17	Female	-	Primary
Pregnant Women (PW)	Tiko	36	Female	Teacher (Primary)	-
Pregnant Women (PW)	Tiko	24	Female	Hairdresser	-
Pregnant Women (PW)	Tiko	22	Female	Fashion designer	--
Pregnant Women (PW)	Tiko	26	Female	Housewife and farmer	-
Pregnant Women (PW)	Tiko	28	Female	Housewife	-
Pregnant Women (PW)	Tiko	32	Female	Petit business	--
Pregnant Women (PW)	Tiko	29	Female	Petit business	-
Pregnant Women (PW)	Bangem	33	Female	Housewife and farmer	-
Pregnant Women (PW)	Bangem	35	Female	Housewife and farmer	-
Pregnant Women (PW)	Bangem	28	Female	Hairdresser	--
Pregnant Women (PW)	Bangem	30	Female	Farmer	-
Pregnant Women (PW)	Bangem	26	Female	Housewife and farmer	-
Pregnant Women (PW)	Bangem	25	Female	Farmer	-

##### Perceived benefits

Participants from all four-district believed that family planning is good and necessary and provided additional explanation in relation to benefits of family planning in relation to the use of contraceptives to prevent pregnancy, promote health and well-being, financial control and minimizes stress, and can protect against other sexually transmitted diseases. Participants provided additional explanation as described below and this is also highlighted in Table 1.

*My partner/ husband, decided to be using condoms so that it will help me take better care of the baby but also prevent unwanted pregnancy for my health and the health of the baby. So, he uses condoms. At times if he wants to go without, he uses the withdrawal method. But he’s very conscious of it*.

[FGD women Tiko/Bangem]

“*As he earlier said, family planning is good because I realized that when you control your bed, you control your finances, and you control your headache. Controlling your headache in the sense that, if you were supposed to have 2 kids, and you were just fooling around anyhow without knowing about family planning. You will have a third one when you are not prepared. The first one may be in class 1 and the second one is in nursery 1. The third one you did not prepare for will come and the financial issues will increase, the headache will also increase. Because you will be paying fees for class 1, nursery 1 then start thinking of buying food for the one the mother is carrying on the back. As I earlier, I will go in for the condom method*”. (FGD, men /Tiko, Ayos).

*I think it is a good thing because even in school, we were thought that family planning is a good thing because it permits to reduce the birth rate between some people. For example, a guy and a girl decide to make a child. The two will first talk to see if it is possible or not with their means. I think that family planning is a good thing. Others think that it is a bad thing because they find it useless and is a wastage of time. Its helps reduce some diseases and permits a good development. That is what I wanted to say*. [FGD men Akonolinga/Tiko].

##### Perceived barriers

Some men reported challenges in communication with women and some reported conflicting interests amongst young adolescent group and the men in their understanding of potential side effects. Some men believed the side effects reported by women in relation to weight gain may hamper the use of family planning. Some of them thinks that it causes infertility and believe that their participation in the discussion was a perfect avenue for them to gain better understanding around miss information and myth in relation to aspects of infertility. See additional information in Table 1.

*“It is necessary that people think of another education on condom because as he have just said, you come across a girl you love and to whom you can’t refuse anything, and she says that she wants something, men are fragile and vulnerable in front of such situations. I think we should complete education on condom because you see there are people who have never seen the female condom which exists since 6 to 7 years. My son told me to leave him alone with this stuff. That the gel is the solution to all these things. I asked him, the action period is how long. He told me to stop disturbing him”.* [FGD Men Tiko/Akonolinga]

*it’s true because at times when you use the condom violently, she tells you how you’re cheating on her. It’s good to talk about it to agree on a method.* [FGD Tiko Men/Akonolinga]

*There are other women in different places that birth to at least 2 children and look for a nurse or a doctor for family planning. They may implant it under her skin or inject it, but when their partner finds out about it, it is a big problem. The one you implant is put in such a way that it can cause damage, we see this at times.* [FGD Tiko Men]

*I am a user of family planning, I started with oral, but when I realized that it causes people to gain weight I moved to implanting and this is my season 2 in implanting. The first 5 years went through, then I went to the second 5 years which is ongoing. Where I live, I generally hear women say it causes ‘internal heat’ some discourage the idea by saying it causes infertility. When I say I’m delighted to be in this discussion or meeting today is that at the end of it all, I should really know the truth about family planning. Whether it causes internal heat or it causes infertility, I need justifications on that one*. [FGD Tiko men]

##### Perceived susceptibility

Some participants reported the vulnerable nature of adolescent girls who are sometimes hesitant to use condom with the notion that it does not provide the pleasure that is deserved. Some men believed this act exposes them to become more vulnerable as they aim to satisfy the girls. This was mostly reported by men who highlighted the need to educate youths and adolescent whom they see as the most vulnerable. On another note, some men reported the need to be caution in the way they use condom and to understand their wives’ menstrual cycle to avoid unintended consequences.

*“Nowadays, what we notice is that most youngsters don’t use condoms. Maybe the boy will want to use the condom, but the girl will refuse saying that the condom reduces the pleasure. So, when the girl says that, you the guy must obey because you want to satisfy your partner. They usually say, “we don’t eat bananas with its skin”. So, to eat a banana you must remove the skin. If you insist, she will refuse. And that is how we go on for it”*. [ Tiko FGD Men].

*“I talk with mine because to be honest, calculating the menstrual cycle is not easy, to this day I cannot say I’ve mastered it. The method I have confidence in is condoms, but we were using a different method, the one for 3 months. She had some issues; she was no longer bleeding so she stopped. After having stopped the side effects creeped up. we saw a doctor who prescribed and proposed some drugs so that her system would return to normal. From there on, her cycle went back to normal, and we are using condoms and practicing abstinence for a year and a half to be certain that her cycle was back to normal”* [FGD Men Akonolinga].

*“Through the course of the process be careful how you carry yourself. If your wife doesn’t shave and you are putting all the pressure, there will be friction and an accident will occur. Or if you don’t understand that your wife shaved 2 days ago there is a likelihood of some ‘shoot out’ you just want to jump on her in excitement immediately it touches a blade it is bound to go off even at the level of wearing it. At times we wear it completely when we are supposed to leave some air at the tip so that it can help in its own way. When you wear it completely, the last thing that touches it, the least friction causes the same accident. Most of these are the modes of how we use condoms”*. [FGD Men Akonolinga/Tiko].

##### Perceived severity

Participants from all four districts expressed their experiences with some of the side effects with the use of family planning methods from both the men and women. Some had doubts and needed additional clarification to get a better understanding due to limited knowledge. Others were more concerned about additional knowledge to motivate them to commence family planning and what method is best.

*“I learned that my girlfriend, has the pill, injection. She experienced some side effects, she was bleeding a lot so because of that we adopted the morning-after pill”*. [FGD men Ayos].

*“My wife took Jadelle for 5 years, and after one year, she started feeling uneasy and had a lot of dizziness, she was getting small illnesses regularly, so we forced to remove it and now we are using condoms, male condoms”*. [FGD Men Tiko].

*“just like me, we started the 3-month injection, but the side effects didn’t permit us to continue so we reverted to male condoms. Tried one that is said to last 5 years. It didn’t work because she was complaining too much, small illnesses weight loss *etc*. so we were forced to…. Now I prefer condoms. I too work at the hospital so when they removed it, they proposed she try another one and she refused”*. [FGD men and women Bangem/Tiko].

An interesting discussion on condoms ranged from the mode of utilization, cost, quality, availability, and ignorance. Some men are of the opinion that though pharmacies may be selling low-quality condoms, it is easier to detect the quality of the condom from the cost. An interesting paradox, as some men estimated the severity of the consequences of using a low-quality condom, others saw it as an equity issue based on affordability because some men are unable to afford a good condom if the quality is determined by price as explained below and in Table 1.

*“Because when you decide to go to the shop for 200cfa. For example, a condom costs 200cfa. Those who are not financially capable, will go for the one for 100cfa”.* [Tiko FGD men].

*“For someone like me who is a local, if we hear rumours that pharmacies are also dealing with local drugs what can I do? Because pharmacies are also looking for cheaper options to make a profit. I, as a local person cannot distinguish good condoms from bad condoms, I just buy them, those are also my worries”.* [FGD men Bangem/Tiko].

*“When they talk about family planning, condoms are part of it, I just want to say that a condom is not supposed to burst. That’s my worry. For the other ones that you implant, the women using them will gain weight. When you use the method then think about the effects, it is concerning”.* [Tiko FGD men]

##### Self-efficacy

Some women think some men are also vulnerable and not trustworthy so important to always use condoms:

*“You could have a stable partner, but if he strays you can still use it, it’s not only to avoid pregnancy. There’re men in the town hall who are married, and they stray, the women can be faithful. When he comes back you don’t know what he has done outside so use the condom, that’s how you’ll avoid illnesses. I don’t joke with that. I have a husband, but I use it”*. [Women Akonolonga FGD].

##### Cues to action

Participants presented various approaches to stimulate the use of family planning which ranges from listening to messages in local languages, educating adolescent and households, dialogue with partners and listening to messages frequently was reported as a motivating factor, especially in a local language.

*“I’m interested, particularly on the part of choice, since there are many side effects there may be a method that suits your wife. It seems many women have that issue so you may need another method but which?”* [FGD men Akonolinga].

*“Since we’re here we have local people around, and all of us are agreeing that you should put messages in the local language, which is pidgin. The way he speaks is different from the way I speak, so when we go back to our communities many people there still have limited education. So pidgin English is good. Therefore, those who can speak English can continue to sensitize in English and those in Pidgin will sensitize in Pidgin”*. [FGD Women Tiko].

*“Because those young ones outside, if they have applications, which help them, she will talk of it to a friend and at the end they will understand the necessity of this application.The beginning will be difficult. When you don’t understand you feel like it is difficult. But it is an ideal idea”.* [FGD men Akonolinga, /Tiko].

Respondents believed that using a digital platform like BornFyne to educate families and households on family planning directly from their phones is an innovative approach. In their opinion, it would help even if the woman or man does not know how to use the mobile phone their children can always train them. They see it as a way of empowering household and not just an individual benefit. In addition to using such platform, they suggest the importance of parent educating their children at home on FP aspects.

*“We understand that an application of this type can educate a mass of people. Because with Android, the phones you can’t buy, which you don’t know of this phone, your daughter can teach you and it understood easily”[FGD Women Ayos/Akonolinga/Bangem].*

*“If the application is limited at the level of education, and what is to be avoided is that I have seen in certain applications where we propose that parents should really emphasize the education of their children, especially young girls. They are vulnerable. We should prompt parents to discuss with their children, boy or girl, we don’t know where the problem can come from”. [ FGD Men Tiko.*

##### Education

Some participants emphasized the need for more education on abstinence and the importance of parents talking with their children at home. One participant listed three key elements in defining contraceptive methods and that the first contraceptive method is education between parents and the child, the second is abstinence which can only be practice with prior education, and the third is the use of condom for protection against sexually transmitted diseases in the event of any risk or unforeseen exposure. CHW reported the need for more education on FP targeted to the men, this was based on their experiences during home visits. They reported that most of the time, FP messages are directed to women during clinic visits and some men are not exposed to this information and sometimes perceived FP as a myth and think that some FP methods can cause infertility.

*“The first contraceptive method is education, parent–child. The second contraceptive is abstinence, and we can’t practice abstinence if we have not been educated. In case of major risk, there is a condom at the third level which is a barrier, especially for STD/STI. It is the first great barrier. There are other contraceptive methods, but a condom is the first barrier”*. [FGD Men Akonolinga/Tiko].

*“In our local areas, we noticed that some people have partners at home and have given birth to more than 2 children, and we suggest they should do something about it, like taking a break. Some women refuse and demand sex”.* [FGD Tiko Men].

*“First, for some people nowadays it’s still a myth, recently during my visits I was talking to a girl who told me that she couldn’t take a method because it would make her not give birth anymore. So, there are some people who don’t even know what family planning is, especially men because in hospitals they talk to only women about it. So, when the woman talks to her husband about it, he’ll even get angry, and it causes problems for the couple. So, it’s difficult to reach a consensus or to agree that we should either take pills or injections. In nearly 300 households that I surveyed, I found about 15 couples who made arrangements together to go to family planning. The second point is that some people want children, and they don’t want to hear things like that because there are families where he’ll tell you that he was with his brother and he’s dead. So, he doesn’t want to hear about family planning”*. [CHW Ayos FGD].

##### Equity concerns in the sensitization of male versus female condoms

Participants expressed an important observation in relation to the use of female condoms. Most men reported that they have never seen a female condom and they do not have an idea what it looks like and how it can be used. They believed that there has been limited sensitization on female condoms and more promotion for male condoms. Some men also reported on the cost of male condoms and the quality of the condoms and their experiences.

*“Another aspect is that we are used to the male condom. Firstly, the male condom is 150 FRS while for females it is not the case. I myself have never seen a female condom. I was surprised that we were talking about it. Even on TV, I have never heard of it. I just hear people talking. We are used to the male condom”.* [FGD Tiko Men].

##### Silo decision making

Most men reported that some women make the decision all by themselves without consulting their partners. In addition, they reported that women go behind and discuss with doctors/nurses and when the side effect becomes serious, then they disclose it to the husband, and sometimes it would be the husband that will observe the effect, as reported below.

*“Some women go behind their partner’s back to see the doctor. Fortunately, some doctors notice this and ask them where their partner is. This is also a challenge in our local community. Some doctors tell the women to rest after giving birth and also insist that the man should rest (stop sexual intercourse). If you force a woman, then that is a different situation. Both men and women need to discuss when they should be sexually active”.[FGD men Akonoliga/Tiko].*

##### Cultural aspects

Other participants provided cultural aspects and practices that they are engaged with and expressed some doubts. One participant reported several encounters with women seeking traditional and herbal treatment to stimulate return to fertility. As a result, participants are curious to understand if the injectable method has an effect on the woman especially during child labor.

*“I know about herbal medicine that can be used to assist most girls. most girls who inject family planning are between 19 and 20 years. At times they come back to me, telling me they have missed their period unsure if it’s because of the injection, because they don’t understand. At times I give them some herbs so that their period comes. I want to know whether it’s the family planning that makes them stop menstruating”*. [FGD Men Tiko/Akonolinga].

*“Second thing, if they inject the one for 5 years and after 5 years they get pregnant, giving birth is an issue. They called me to come during labour. I didn’t reach the hospital, but I have a ‘stick’ I give to women who are in labour and between 10 and 15 min that child will be born. After this time, it will only be operation. I don’t really know whether that injection affects you after expiring if you get pregnant it will cause issues during delivery. Most girls in my area call me on the phone asking me to see them/come to the hospital. I help them the way my father showed me, I just don’t know whether those 2 things, the injection they take makes them skip menstruation and the time it has expired, and you are in labour. It disturbs them”* [FGD Men Tiko/Ayos].

Generally, the men were very pleased with the focus group discussion and the topic of the discussion, and this was reported across all four districts.

*“My comment is that I am happy about this discussion and all my seniors and juniors are happy about this meeting. We don’t want this meeting to stop today because everyone is happy. It is 23 years since we haven’t had such a meeting. We are heirs in this village, born, grown, aged, look at the class of men we already have. So, this meeting is hearted. The day you need us we can include more people for us to be more numerous. The elders asked me to talk as such”*. [Men Akonolinga FGD].

##### Identifying the family planning messages to address gaps and unmet needs from participants’ responses

Table 1 presents the identified knowledge gaps based on participants' responses as described in the analysis section above. These knowledge gaps range from their need to understand when to use a family planning method as described in the excerpts above, and when to remove it before and or after giving birth and including post-partum. Most importantly, what type of family planning is best and how to go about it especially removing or deciding to stop. Participants need to understand the potential short- and long-term side effects of family planning methods. They expressed the need to break communication barriers and encourage education and family-oriented discussions, especially targeting youth and adolescent girls and boys. We categorized these knowledge gaps and identified intervention messages that aligns with the gaps from various sources [26–37] (https://www.doctissimo.fr/html/sante/mag_2001/mag0427/sa_3940_nexplanon.htm#comment-se-passe-le-retrait-de-l-implant) and presented in Table 2. Table 2 list the identified gaps from the participant’s responses and the patterns that emerged from the data, including excerpts from participants that align to the identified gap. The last column presents sample messages from the literature that aligns with identified gaps. Thus, the next step present how the sample messages were further contextualized with the community, and validated, to be uploaded into the platform.

Other aspects considered in the identification process of the gaps are listed below.

1. Are there existing guidelines for FP within each district or health facility?
2. language used in communicating family planning messages in each district.
3. The person communicating the message.
4. Channel of communication.

##### Contextualizing family planning messages for the BornFyne-PNMS version 2.0

The evaluation of messages across diverse literacy levels and gender groups in all four districts revealed distinct patterns. Among women unable to read and write, 48% found the messages challenging, while 52% understood the messages well. In contrast to that, women with reading and writing skills displayed a higher understanding, with 56% deeming the messages well or perfectly understandable. Similar trends were observed among men, where 46% of those unable to read or write struggled, didn't understood the messages well. Men with literacy skills exhibited an 80% understanding rate, emphasizing the positive impact of literacy on message comprehension. These results underscore the importance of tailoring communication strategies based on literacy levels and gender differences for more effective outreach.

Of the 50 messages that were tested, 24 messages were revised as shown in Table 8 and Supplemental Table 1. The final messages uploaded into the BornFyne platform are presented in Table 8 and supplemental table 1. The final messages were also submitted to the Department of Family Health for final review and validation. The messages are translated into French, pidgin English and Beti languages. The French version of the Supplemental Table 1 is available upon request. Generally, men presented a better understanding of the content of the messages compared to women. However, the messages were understood by the participants, but some key words used was a concern from participants and they proposed the importance of changing the wordings with common word or synonym as shown in Table 8 and Supplemental Table 1. Words underlined in the messages signify terms identified during the FGD testing that required rephrasing. The community members proposed replacements for these underlined words to better align with their understanding and that of their community.

**Table 8:** Contextualizing family planning messages for the BornFyne-PNMS version 2.0

Intervention messages from WHO, USAID, UNFPA etc.	**Contextualizing the messages**		
**1. What is family planning? (underlined words are the recommended words that needed to be reworded)**	**Female responses during FGD (highlighted words are the proposed words from female respondents)**	**Male responses during FGD (highlighted words are the proposed words from male respondents)**	**Revised messages with participants during FGD to be uploaded into BornFyne-v2.0**
Family planning allows young girls and boys, women, men and couples to determine the number and timing of pregnancies through the use of a method of contraception [20]	Family planning allows young girls and boys, women, men and couples ***to know*** the number and timing of pregnancies through the use of a method of contraception	Family planning allows young girls and boys, women, men and couples ***to decide the number*** and timing of pregnancies through the use of a method of ***family planning***	Family planning allows young girls and boys, women, men and couples to know and decide the number and timing of pregnancies through the use of a method of family planning
**2. Importance of family planning**	**Female responses during FGD**	**Male responses during FGD**	**Revised messages with participants during FGD**
- Why is family planning important?			
**It allows the mother ** [20]			
to rest between two pregnancies	to rest between two pregnancies	to rest between two pregnancies	to rest between two pregnancies
to take care of the newborn	to take care of the newborn	to take care of the newborn	to take care of the newborn
to keep his health to take care of the child and the rest of the family	to keep his health to take care of the child and the rest of the family	to keep his health to take care of the child and the rest of the family	to keep his health to take care of the child and the rest of the family
to be able to carry out their own activities[ref]	to be able to carry out their own activities[ref]	to be able to carry out their own activities[ref]	to be able to carry out their own activities[ref]
**to the father ** [20, 21]			
to protect the health of the mother	to protect the health of the mother	to protect the health of the mother	to protect the health of the mother
to be able to receive more attention from the mother	to be able to receive more attention from the mother	to be able to receive more attention from the mother	to be able to receive more attention from the mother
better plan family expenses	better plan family ***income***	better plan family **income**	better plan family income
better support children	better support children	better support children	better support children
**to the child ** [20]			
to benefit from all the affection of mom and dad	to benefit from all the ***love*** of mom and dad	to benefit from all the **love** of mom and dad	to benefit from all the love of mom and dad
to have time to feed exclusively at the mother’s breast	to have time to feed **only** at the mother’s breast	to have time to feed **only** at the mother’s breast	to have time to feed only at the mother’s breast
to be in good health and better protect	to be in good health and better protect	to be in good health and better protect	to be in good health and better protect
to be better educated	to be better educated	to be better educated	to be better educated

## Discussion

This study used the HBM to understand the community’s level of knowledge and perspective on FP, as well as the use and potential side effects of contraceptives including post-partum. The study found that most of the community members across the four districts have heard of FP, and most of them have some level of understanding about FP which corroborates with the national statistics from the Ministry of Public Health [7, 8]. However, there exist important knowledge gaps and unmet needs within the community that require additional intervention, particularly through education and sensitization. Most importantly, most men and women have not seen a female condom and do not understand how they are used. Adolescent and youth ignorance and level of knowledge was reported as an important aspect that required parental intervention.

The survey responses and FGD converge to illuminate an understanding of family planning dynamics in the diverse districts. While the quantitative surveys demonstrate demographic patterns, educational backgrounds, and preferences including possible reported side effects, the FGDs inquire complexities of participants’ lived experiences, beliefs, and challenges related to family planning. Survey data reveals a predominant awareness of family planning, with marked district-specific variations in media preferences for disseminating information. The FGDs, in turn, shed light on the complexity of decision-making processes, particularly emphasizing the challenges faced by men in communication with their partners. Perceived benefits, such as economic stability and health concerns, cut through both survey responses and FGDs, underscoring a shared recognition of the advantages of family planning. Concerns about contraceptive methods, especially their side effects and cultural beliefs, were prominent in the FGDs, offering qualitative explanations to the quantitative findings. The intersection of survey results and FGD narratives emphasizes the importance of tailored communication strategies that address district-specific preferences while navigating cultural nuances and fostering inclusive discussions about family planning within households. For example, respondents of household survey preferred languages to listen to FP messages was French and English, which aligns to what is predominantly spoken in their respective districts, but the FGDs reveal additional insights on preference in the use of local dialects. The synthesis of these datasets provides a comprehensive lens, enriching our comprehension of family planning dynamics beyond mere statistics to encompass the contextual complexities of individuals’ perspectives and experiences.

### Education

Most participants especially men reported the need for more education at different levels. They highlighted the key role of education between the parents and children as an attribute in defining contraceptive methods. This education is directed not only to adolescent, but extended to men. Men reported the need to educate the adolescents whom they considered vulnerable. Men were of the opinion that there is a need for more education and sensitization on FP methods, contraceptive choices and side effects. With a focus on men because women get more information on family planning when they visit the health facility, but some men are still ignorant and need to be sensitized.

#### Strategies and communication channels for family planning

Understanding the varied channels through which participants receive information on family planning is crucial for tailoring effective outreach strategies. The survey results shed light on the primary sources of family planning messages across different districts, revealing distinct patterns.The majority of participants across all districts reported health facilities as the primary source of family planning messages. Notably, Tiko emerged as the district with the highest reliance on health facilities, followed by Ayos, Bangem, and Akonolinga. This underscores the pivotal role that healthcare institutions play in disseminating information and promoting awareness. Following health facilities, CHW were identified as another significant source of FP messages. Tiko again led in this category, indicating the effectiveness of community-level engagement. Ayos, Bangem, and Akonolinga followed suit, with varying degrees of reliance on community health workers for family planning information. Television emerged as a notable source of family planning messages, with Tiko exhibiting the highest dependence on this medium. Ayos and Akonolinga also reported television as a substantial source, while Bangem showed comparatively less reliance. An understanding of this exisitng sources of FP messages in combinaiton of participants responses on preferences in listening to FP messages from their mobile phones and the frequency provides a deeper undertsnading into the importance of establishing a continous educational platform to compliment existing sources and support communities and households in improving their knowledge of FP and increase awareness and uptake.

### Variations in FP information sources

The visual impact of television in conveying family planning messages is evident, especially in districts with higher engagement. The influence of interpersonal relationships on family planning awareness was evident, with most participants citing family and friends as a source of information. Leveraging social networks for disseminating information could prove beneficial, given the demonstrated influence of personal connections. Radio, a traditional yet potent medium, played a role in family planning awareness. Ayos, Bangem, and Akonolinga followed suit, indicating the continued relevance of radio as a channel for reaching diverse audiences. The accessibility and widespread reach of radio make it an essential tool for communication campaigns. Recognizing the community dynamics and incorporating innovative digital platforms as potential spaces for information dissemination could enhance outreach efforts for FP messages and improved knowledge [13, 14]. These findings underscore the diversity in channels through which family planning messages can reach communities in different districts. Most respondents from the survey expressed willingness to listen to famly plannig regularly from their phone and this was elaborated in the FGD with interest in local languages.

### Generating tailored FP messages for specific groups based on unique needs

Tailoring communication strategies to maximize the impact of preferred channels, while addressing specific community dynamics, is crucial for ensuring the success of family planning initiatives [16, 17]. Some men reported important barriers in communication with women which sometimes hinder discussions about family planning. In the same way, some women reported challenges in discussing family planning with their partners and would prefer to engage in family planning without their knowledge. This component causes misunderstanding as some men reported that women would engage in family planning and when the side effects get worse on them that is when the men will realize they had been practicing family planning.

In rural areas, the priority is reaching the most remote households and identifying specific gaps to inform content contextualization process and generate tailored interventional messages that align with the community’s need. It’s important to note that family planning is not entirely unfamiliar to the community; according to the household survey, most households have heard of family planning, and some have utilized it. However, the objective is to comprehend and pinpoint the existing gaps, strategizing on effective interventions that can address these gaps. Thus, our approach involves addressing content-related gaps to improve knowledge and promote FP adoption and practices. We believed that our approach can be use not only to contextualize existing FP content, but also in developing new content for FP messages. In this process, we observed a breadth of existing interventional FP messages as listed in references [26–37] (https://www.doctissimo.fr/html/sante/mag_2001/mag0427/sa_3940_nexplanon.htm#comment-se-passe-le-retrait-de-l-implant) from varied sources in addition to the Ministry of Public Health. Instead of reinventing the wheel in trying to develop new content, we identified existing messages that align with the identified gaps and then contextualize the message. The key aspect to note in this process is to ensure the messages are contextualized with the community and validated by both the community in question and the authorities. Thus, we believed that in the absence of existing interventional FP messages that are contextualized and align to a specific identified gap, our approach can help inform the development of any new FP content to address an identified gap.

### The use of effective FP communication strategies and appropriate medium of communication

For effective communication about family planning, it is necessary to explore various methods of communication. Most communication strategies are television and radio, as reported in the survey. Most household also reported health facilities as main sources. However, not all women are able to attend ANC when they are pregnant due to cost and other cultural barriers. In addition, some women who attend ANC may not obtain a better understanding of the information communicated to them due to language barriers and their inability to properly process the information. As suggested by some of the men who participated in the FGDs, there is a need for a holistic approach for communication in households and should start with the parents in addition to other methods to target households and youth. There is a need to utilize innovative approaches like the BornFyne FP feature that can continuously sensitize communities and households using various langauges and motivate participants to engage into FP practices. Addressing unmet needs of FP requires careful identification of the knowledge gaps within the community especially the timing of the use of family planning, their understanding of FP methods and potential side effects. This is important because post-partum mothers’ needs are different from preconception mothers and also adolescents. In addition, the potential side effects of some of the contraceptive methods are often misrepresented by some women and it all depends on how they’ve been educated and sensitized.

### Preference and frequency in listening to FP messages

The preferences for listening to FP messages on a varied frequency, such as weekly, monthly, or daily, through platforms like BornFyne, signify the importance of adaptable communication strategies catering to diverse preferences. The substantial demand for weekly messages, particularly in Tiko, aligns with studies emphasizing the significance of consistent and regular health communication [38] and the role of using digital platforms to support educational messages [13, 14]. This aligns with findings from similar studies, such as a community-based intervention in Nigeria, where frequent and sustained messaging positively influenced FP knowledge and uptake [39]. The inclination towards diverse frequencies suggests the need for personalized approaches, aligning with the literature on tailored health interventions [40]. The BornFyne platform and its FP feature aligns with studies highlighting the effectiveness of mobile health applications in enhancing health knowledge and behaviours [13, 14, 41]. The emphasis on messages in local dialects aligns with the literature emphasizing the importance of culturally sensitive health communication [42, 43]. Overall, these results highlight the potential effectiveness of BornFyne FP feature in addressing FP knowledge gaps, drawing parallels with similar studies, and reinforcing the importance of culturally tailored, consistent, and flexible health communication strategies.

### The language use in communicating FP messages

The utilization of BornFyne, delivering family planning messages in local dialects, aligns accordingly with the FP2030 agenda and Cameroon’s commitment to addressing unmet needs in family planning [8]. By tailoring communication strategies to diverse communities, BornFyne addresses knowledge gaps and preferences identified through surveys and focus group discussions. This approach not only can increase awareness, understanding, and acceptance of family planning methods, but also empowers households, especially women and adolescents, with accessible and culturally sensitive information. Such initiatives resonate with global efforts to enhance reproductive health services, aligning with FP2030’s goal of reaching more women and girls with quality family planning services. The emphasis on including all parties and addressing specific regional preferences, as evidenced in the survey and FGD results, contributes to advancing Cameroon's FP2030 agenda by fostering informed decision-making and facilitating open dialogues on family planning in Cameroon and beyond [8].

As the family planning messages are uploaded into the BornFyne-PNMS, the next step will test the interventional messages with a small group of participants to evaluate the potential impact of the intervention messages and areas for adjustment and refinement.

Acknowledging the previously mentioned goals of the FP2030 agenda in Cameroon and the corresponding objectives of the BornFyne FP feature, which are in harmony with these outlined aims, there is a need to revise FP guidelines in Cameroon. This revision will ensure a systematic approach in delivering FP services to enhance overall uptake. Consequently, the BornFyne team has collaborated with the Ministry of Public Health, specifically the Department of Family Health, to support initiatives aimed at enhancing FP awareness and uptake. The subsequent phase involves and incorporating the WHO digital adaptation kit for FP into the BornFyne family planning feature and content adaptation.

## Strengths and limitations

The study provides valuable insights into family planning knowledge, preferences, and perceptions in diverse districts of Cameroon. The combination of quantitative survey data and qualitative focus group discussions enhances the comprehensiveness of the findings, offering a nuanced understanding. The large survey sample size across multiple districts adds robustness to the study’s result, and the application of the Health Belief Model provides a theoretical framework for interpreting the results and informing interventions. While the survey and focus group discussions provided valuable insights, potential limitations include self-reporting bias, as participants may offer socially desirable responses. The approach is useful and replicable process to adapt and contextualize FP messages and communication channels to a local population and its relevance to practice. However, there are some limitations to the study. The study’s cross-sectional nature limits the ability to establish causation or assess changes over time. Additionally, the findings may not be fully generalizable beyond the specific districts studied and the limitation of a statistical powered sampling limits the generalizability of the results to the specific population targeted, Potential limitation in the survey responses due to reporting bias and selection of household respondents. The use of social marketing team and strategies is a strength to this study as it allows effective consideration of equity components and contextualizing messages that meets the needs of the community tailored to their needs and preferences.

## Conclusion

Developing effective FP intervention messages requires a nuanced understanding of community perspectives. The BornFyne-PNMS family planning feature, informed by the Health Belief Model, addresses knowledge gaps by delivering educational messages in local dialects via mobile phones. The study’s findings underscore the importance of community-based approaches to FP education, targeting specific populations with tailored messages to promote awareness, acceptance, and informed decision-making. The validated messages were incorporated into BornFyne-family planning module.

### Supplementary Information

Supplementary Material 1.

## Data Availability

The authors confirm that all data generated or analysed during this study are included in this published article in the main manuscript and as Supplemental file. The French version of the Supplemental file 1 is available upon request.

## References

[1] Prata N, Weidert K, Sreenivas A. Meeting the need: youth and family planning in sub-Saharan Africa. Contraception. 2013;88(1):83–90. 23177267 10.1016/j.contraception.2012.10.001

[2] Ahmed S, Li Q, Liu L, Tsui AO. Maternal deaths averted by contraceptive use: an analysis of 172 countries. Lancet. 2012;380(9837):111–25. 22784531 10.1016/S0140-6736(12)60478-4

[3] World Health Organization. Family planning/contraception. Geneva: World Health Organization; 2018. Available from: https://www.who.int/news-room/fact-sheets/detail/family-planning-contraception. Accessed Dec 2023.

[4] Potts M. Family planning is crucial to child survival. Newt Res Triangle Park N C. 1990;11(4):2. 12283718

[5] Countdown 2015 Europe. Family planning & women’s empowerment. https://www.countdown2030europe.org/storage/app/media/IPPF_Factsheet-1_Empowerment.pdf. Accessed Aug 2023.

[6] United Nations, Department of Economic and Social Affairs, Population Division. Trends in contraceptive use worldwide. New York: United Nations; 2015. Available from: https://www.un.org/en/development/desa/population/publications/pdf/family/trendsContraceptiveUse2015Report.pdf. Accessed Nov 2023.

[7] Institut National de la Statistique (INS) et ICF. Planification familiale. In: Enquête Démographique et de Santé et à Indicateurs Multiples 2011. Calverton; 2011. p. 99–117. Available from: https://dhsprogram.com/pubs/pdf/FR260/FR260.pdf. Accessed Dec 2023.

[8] FP2030 Cameroon Available at: https://www.fp2030.org/cameroon/documents/#tab. Accessed Dec 2023

[9] Egbe TO, Atashili J, Talla E, et al. Effect of performance-based financing home visiting on the use of modern methods of contraception in the kumbo east health district, Cameroon. Contracept Reprod Med. 2016;1:19. 10.1186/s40834-016-0030. 29201408 10.1186/s40834-016-0030 PMC5693568

[10] WHO, UNICEF, UNFPA, World Bank Group, and the United Nations Population Division. Trends in maternal mortality: 1990 to 2015. Geneva: World Health Organization; 2015. Available from: http://apps.who.int/iris/bitstream/handle/10665/194254/9789241565141_eng.pdf;jsessionid=290616ED3134931C478D30A44F1A614A?sequence=1. Accessed 29 Sept 2023.

[11] Cameroon 2018 Demographic and Health Survey Report. Available at https://dhsprogram.com/pubs/pdf/SR266/SR266.pdf. Accessed 20 Apr 2024.

[12] Nansseu JRN, Nchinda EC, Katte J, Nchagnouot FM, Nguetsa GD. Assessing the knowledge, attitude and practice of family planning among women living in the Mbouda health district, Cameroon. Reprod Health. 2015;12:92. 26452643 10.1186/s12978-015-0085-9 PMC4598975

[13] Chukwu E, Gilroy S, Addaquay K, Jones NN, Karimu VG, et al. Formative study of mobile phone use for family planning among young people in Sierra Leone: global systematic survey. JMIR Form Res. 2021;5(11):e23874. 10.2196/23874. PMID:34766908;PMCID:PMC8663572. 34766908 10.2196/23874 PMC8663572

[14] Johnson D, Juras R, Riley P, Chatterji M, Sloane P, et al. A randomized controlled trial of the impact of a family planning mHealth service on knowledge and use of contraception. Contraception. 2017;95(1):90–7. 10.1016/j.contraception.2016.07.009.S0010-7824(16)30163-9. 27421767 10.1016/j.contraception.2016.07.009.S0010-7824(16)30163-9

[15] Kouyate AR, Nash-Mercado A. A guide for developing family planning messages for women in the first year postpartum. Baltimore: ACCESS-FP; 2010.

[16] Mwaikambo L, Speizer IS, Schurmann A, Morgan G, Fikree F. What works in family planning interventions: a systematic review. Stud Fam Plan. 2011;42(2):67–82 http://www.jstor.org/stable/41310713. 10.1111/j.1728-4465.2011.00267.x PMC3761067 21834409

[17] Teklemariam EY, Getachew S, Kassa S, Atomsa W, Setegn M. What works in family planning interventions in Sub-Saharan Africa: a scoping review. J Womens Health Care. 2019;8:471. 10.35248/2167-0420.19.8.471. 10.35248/2167-0420.19.8.471

[18] Nkangu MN, Okwen PM, Mbuagbaw L, et al. A protocol for a pilot cluster randomized control trial of e-vouchers and mobile phone application to enhance access to maternal health services in Cameroon. Pilot Feasibility Stud. 2020;6:45. 10.1186/s40814-020-00589-y. 32313683 10.1186/s40814-020-00589-y PMC7155248

[19] Obegu P, Nkangu M, Ngo NV, Wanda F, Kasonde M, Kibu OD, et al. Community participation for reproductive, maternal, newborn and child health: insights from the design and implementation of the BornFyne-prenatal management system digital platform in Cameroon. Front Digit Health. 2023;17(5):1218641. 10.3389/fdgth.2023.1218641. PMID:37664872;PMCID:PMC10470631. 10.3389/fdgth.2023.1218641 PMC10470631 37664872

[20] Nkangu M, Njoache MN, Obegu P, Wanda F, Ngo VN, Fantaye A, et al. Developing the BornFyne prenatal management system version 2.0: a mixed method community participatory approach to digital health for reproductive maternal health. Oxford Open Digit Health. 2024;2:oqae012. 10.1093/oodh/oqae012. 10.1093/oodh/oqae012 PMC11932395 40230970

[21] Rosenstock IM, Strecher VJ, Becker MH. Social learning theory and the health belief model. Health Educ Q. 1988;15(2):175–83. 10.1177/109019818801500203. PMID: 3378902. 3378902 10.1177/109019818801500203

[22] Becker MH. The health belief model and sick role behavior. Health Educ Monogr. 1974;2(4):409–19. 10.1177/109019817400200407. 10.1177/109019817400200407

[23] World Bank Population Data. Available online: https://data.worldbank.org/indicator/SP.POP.TOTL?locations=CM. Accessed 10 March 2022.

[24] de Walque D, Robyn PJ, Saidou H, Sorgho G, Steenland M. Looking into the performance-based financing black box: evidence from an impact evaluation in the health sector in Cameroon. Health Policy Plan. 2021;25:835–47. 10.1093/heapol/czab002. PMID: 33963406. 10.1093/heapol/czab002 PMC12147217 33963406

[25] Cameroon launched Universal Health Coverage Phase I. Available at: https://cameroonnewsagency.com/first-phase-of-universal-health-coverage-launched-cameroonians-urged-to-register-massively/. Accessed Jul 2023.

[26] Archive: Knowledge 4 Health tool kit. Available at: https://toolkits.knowledgesuccess.org/sites/default/files/family_planning_reflections_fgd_guide-eng.pdf. Accessed May 2023.

[27] Family Planning Handbook. Available at: https://fphandbook.org/welcome-fphandbookorg. Accessed May 2023.

[28] Zavodny M. Fertility and parental consent for minors to receive contraceptives. Am J Public Health. 2004;94(8):1347–51. 10.2105/ajph.94.8.1347. Erratum.In: Am J Public Health.2005Feb;95(2):194. PMID:15284042; PMCID: PMC1448454. 15284042 10.2105/ajph.94.8.1347 PMC1448454

[29] Plan International. Available at: https://www.plan-international.fr/nos-combats/sante-sexualle-et-reproductive/causes-et-consequences-des-grossesses-precoces/. Accessed May 2023.

[30] Acteur de ma Sante. Available at: https://acteurdemasante.lu/fr/sante-bien-etre-de-la-femme/le-sterilet-tout-savoir-sur-le-dispositif-intra-uterin-au-cuivre/. Accessed May 2023.

[31] Contraception and family planning. Available at: https://www.who.int/news-room/questions-and-answers/item/coronavirus-disease-covid-19-contraception-and-family-planning. Accessed Apr 2023.

[32] Elsan. Impant Contraceptive. Available at: https://www.elsan.care/fr/pathologie-et-traitement/diseases-gynecological/implant-contraceptif-definition-pose. Accessed May 2023.

[33] Le Manuel MSD. Available at: https://www.msdmanuals.com/fr/professional/gyn%C3%A9cologie-et-obst%C3%A9trique/planning-familial/dispositif-intra-ut%C3%A9rin. Accessed May 2023.

[34] Family Planning handbook. Available at: https://fphandbook.org/questions-and-answers-about-intrauterine-device. Accessed Apr 2023.

[35] Sante bien de la femme-Contraception. Available at: https://acteurdemasante.lu/fr/sante-bien-etre-de-la-femme/contraception-hormonale-avantages-et-inconvenients-des-pilules-microprogestatives/. Accessed Apr 2023.

[36] CNGOF 2018 Contraception. Available at: http://www.cngof.fr › menu-contraceptionAccessed Apr 2023.

[37] United Nations. Population division: world population prospects 2019. Available from: https://population.un.org/wpp/. Accessed 30 Jul 2023.

[38] Oyeyemi SO, Wynn R. Giving cell phones to pregnant women and improving services may increase primary health facility utilization: a case control study of a Nigerian project. Reprod Health. 2014;11(1):8. 24438150 10.1186/1742-4755-11-8 PMC3898403

[39] Manganello J, Gerstner G, Pergolino K, Graham Y, Falisi A, Strogatz D. The relationship of health literacy with use of digital technology for health information: implications for public health practice. J Public Health Manag Pract. 2017;23(4):380–7. 26672402 10.1097/PHH.0000000000000366

[40] Ijadunola MY, Abiona TC, Ijadunola KT. A review of family planning studies in Nigeria. Afr J Reprod Health. 2010;14(3):17–26. 21812197

[41] Kreuter MW, Strecher VJ, Glassman B. One size does not fit all: The case for tailoring print materials. Ann Behav Med. 2000;22(4):276–83. 10721433 10.1007/BF02895958

[42] Feroz A, Perveen S, Aftab W. Role of mHealth applications for improving antenatal and postnatal care in low- and middle-income countries: a systematic review. BMC Health Serv Res. 2017;17(1):704. 29115992 10.1186/s12913-017-2664-7 PMC5678803

[43] Holt CL, Roberts C, Scarinci I, Wiley SR, Eloubeidi M, et al. Development of a spiritually based educational program to increase colorectal cancer screening among African American men and women. Health Commun. 2014;29(5):451–60. 10.1080/10410230903023451 19657823

