# Supplementary material for: Contextualizing family planning messages for the BornFyne-PNMS digital platform in Cameroon: a community-based approach
Source: Reprod Health. 2024 Aug 26;21:124. doi: 10.1186/s12978-024-01842-w (PMC11346027; doi:10.1186/s12978-024-01842-w)
Supplement: Supplementary file 1 — Supplementary Material 1. [file 12978_2024_1842_MOESM1_ESM.doc]

**Supplemental Table 1: Contextualizing family planning messages for the BornFyne-PNMS version 2.0**

| Intervention messages from WHO, USAID, UNFPA etc. | **Contextualizing the messages** | | |
| --- | --- | --- | --- |
| **1. What is family planning? (underlined words are the recommended words that needed to be reworded)** | **Female responses during FGD**  **(highlighted words are the proposed words from female respondents)** | **Male responses during FGD**  **(highlighted words are the proposed words from male respondents)** | **Revised messages with participants during FGD to be uploaded into BornFyne-v2.0** |
| Family planning allows young girls and boys, women, men and couples to determine the number and timing of pregnancies through the use of a method of contraception ^20^ | Family planning allows young girls and boys, women, men and couples ***to know*** the number and timing of pregnancies through the use of a method of contraception. | Family planning allows young girls and boys, women, men and couples ***to decide the number*** and timing of pregnancies through the use of a method of ***family planning*** | Family planning allows young girls and boys, women, men and couples to know and decide the number and timing of pregnancies through the use of a method of family planning |
|  |  |  |  |
| **2. Importance of family planning** | **Female responses during FGD** | **Male responses during FGD** | **Revised messages with participants during FGD** |
| - Why is family planning important? |  |  |  |
| **It allows the mother ^20^** |  |  |  |
| to rest between two pregnancies | to rest between two pregnancies | to rest between two pregnancies | to rest between two pregnancies |
| to take care of the newborn | to take care of the newborn | to take care of the newborn | to take care of the newborn |
| to keep his health to take care of the child and the rest of the family | to keep his health to take care of the child and the rest of the family | to keep his health to take care of the child and the rest of the family | to keep his health to take care of the child and the rest of the family |
| to be able to carry out their own activities. | to be able to carry out their own activities[ref] | to be able to carry out their own activities[ref] | to be able to carry out their own activities[ref] |
|  |  |  |  |
| **to the father ^20,21^** |  |  |  |
| to protect the health of the mother | to protect the health of the mother | to protect the health of the mother | to protect the health of the mother |
| to be able to receive more attention from the mother | to be able to receive more attention from the mother | to be able to receive more attention from the mother | to be able to receive more attention from the mother |
| better plan family expenses | better plan family ***income*** | better plan family **income** | better plan family income |
| better support children | better support children | better support children | better support children |
|  |  |  |  |
| **to the child ^20^** |  |  |  |
| to benefit from all the affection of mom and dad | to benefit from all the ***love*** of mom and dad | to benefit from all the **love** of mom and dad | to benefit from all the love of mom and dad |
| to have time to feed exclusively at the mother's breast | to have time to feed **only** at the mother's breast | to have time to feed **only** at the mother's breast | to have time to feed only at the mother's breast |
| to be in good health and better protect | to be in good health and better protect | to be in good health and better protect | to be in good health and better protect |
| to be better educated | to be better educated | to be better educated | to be better educated |
|  |  |  |  |

| **3. When to start and how to stop family planning** | **Female responses during FGD** | **Male responses during FGD** | **Revised messages with participants during FGD** |
| --- | --- | --- | --- |
| **- When to get pregnant?** |  |  |  |
| Ideally, not before the age of 18, to avoid complications due to early pregnancy and childbirth in young girls aged 15 to 19 **^20^** | ***Normally***, not before the age of 18, to avoid complications due to early pregnancy and childbirth in young girls aged 15 to 19. | ***Normally***, not before the age of 18, to avoid complications due to early pregnancy and childbirth in young girls aged 15 to 19. | Normally, not before the age of 18, to avoid complications due to early pregnancy and childbirth in young girls aged 15 to 19. |
| -   **When to start family planning?** |  |  |  |
| For the woman who has just given birth, it is advisable, immediately after childbirth, to use a method of family planning to avoid pregnancy. If you do not practice exclusive breastfeeding immediately after delivery, it is advisable to use another form of contraception **^20^** | For the woman who has just given birth, it is advisable, immediately after childbirth, to use a method of family planning to avoid pregnancy. If you do not practice only breastfeeding immediately after delivery, it is advisable to use another form of contraception. | For the woman who has just given birth, it is advisable, immediately after childbirth, to use a method of family planning to avoid ***Complications that may occur***. If you do not practice ***only*** breastfeeding immediately after delivery, it is advisable to use another form of contraception. | For the woman who has just given birth, it is advisable, immediately after childbirth, to use a method of family planning to avoid Complications that may occur. If you do not practice only breastfeeding immediately after delivery, it is advisable to use another form of contraception. |
| For the girl, from the onset of menstruation. Parental consent is mandatory ^22^. But ideally from 15 years old. | For the girl, ***from the start of the period.you will have to must dis***cuss it with your parents. But ***normally***, from 15 years old | For the girl, from the onset of their period, consent from the parents is a must. But normally from 15 years old. | For the girl, from the start of their period, you must discuss it with your parents. But normally from 15 years old. |
| - Risk of early pregnancy? |  |  |  |
| Exposure to cervical cancer | Uterus or ***cancer of the womb*** | Exposure to ***women's cancer*** | Exposure to cancer of the womb |
| Exposure to STIs and HIV-AIDS ^23^ | Women illnesses | Exposure to passé passé (gonnorhoea) and HIV-AIDS | Exposure to women's illnesses and gonorhoea/HIV |
| Exposure to abortions and their consequences ^23^ | Exposure to abortions and their consequences | Exposure to abortions and their consequences | Exposure to abortions and their consequences |
|  |  |  |  |
| - **How and When to remove the implant or the** Intra intra-uterine device **(IUD) (T) ^24^?** |  |  |  |
| If you use an implant, you can remove it at any time (maximum 3 years after placement) **^24^** | If you use an implant, you can remove it at any time (maximum 3 years after placement) | If you use an implant, you can remove it at any time (maximum 3 years after placement) | If you use an implant, you can remove it at any time (maximum 3 years after placement) |
| If you use the IUD or the “T”, you can remove it at any time (up to 10 years after insertion) ^25^ | If you use the IUD or the copper “T”, you can remove it at any time (up to 10 years after insertion) . | If you use the IUD or the copper “T”, you can remove it at any time (up to 10 years after insertion). | If you use the IUD or the copper “T”, you can remove it at any time (up to 10 years after insertion) . |
| NB: They can only be removed by a trained healthcare provider ^26^ | They can only be removed by a trained healthcare provider. | They can only be removed by a trained healthcare provider. | They can only be removed by a trained healthcare provider. |
|  |  |  |  |
| **How long does the effect of the implant and the IUD or “T” last in the body?** ^27^ |  |  |  |
| The contraceptive effect of the implant ends three weeks later after its removal. The implant may decrease or eliminate bleeding or, conversely, cause disordered/irregular bleeding **^27^** | The contraceptive effect of the implant ends three weeks later after its removal. The implant ***may stop bleeding*** or, ***it can cause*** disordered/irregular bleeding. | The contraceptive effect of the implant ends three weeks later after its removal. The implant ***may stop bleeding*** or, ***it can cause*** disordered/irregular bleeding. | The contraceptive effect of the implant ends three weeks later after its removal. The implant may stop bleeding or, it can cause disordered/irregular bleeding. |
| The contraceptive effect of the IUD or the "T" ends immediately after its removal ^27^ | The contraceptive effect of the IUD or the "T" ends immediately after its removal. | The contraceptive effect of the IUD or the "T" ends immediately after its removal. | The contraceptive effect of the IUD or the "T" ends immediately after its removal. |
| Late withdrawal of long-acting methods, such as implants or the intrauterine device (IUD) , does not pose any medical problem ^28^ | Late withdrawal of long-acting methods, such as implants or the intrauterine device (IUD), does not ***cause*** any medical problem | Late withdrawal of long-acting methods, such as implants or the intrauterine device (IUD), does not ***cause*** any medical problem | Late withdrawal of long-acting methods, such as implants or the intrauterine device (IUD), does not cause any medical problem |
| NB: Do not try to remove the contraceptive method yourself; wait until you have access to health care from a qualified provider ^26^ | Do not try to remove the contraceptive method yourself; wait until you have access to health care from a qualified provider. | Do not try to remove the contraceptive method yourself; wait until you have access to health care from a qualified provider. | Do not try to remove the contraceptive method yourself; wait until you have access to health care from a qualified provider. |

| **4. Management after implant or IUD removal** | **Female responses during FGD** | **Male responses during FGD** | **Revised messages with participants during FGD** |
| --- | --- | --- | --- |
| - How to manage the transition process |  |  |  |
| After the implant or IUD has been removed for any reason and you do not want to get pregnant at that time, you should immediately start using another method of birth control, such as condoms. Women may experience mild discomfort and slight bleeding under the skin after an implant removal. **^29^** | After the implant or IUD has been removed for any reason and you do not want to get pregnant at that time, you should immediately start using another method of birth control, such as condoms. Women may experience **slight pain** and slight bleeding under the skin after an implant removal | After the implant or IUD has been removed for any reason and you do not want to get pregnant at that time, you should immediately start using another method of birth control, such as condoms. Women may experience ***slight pain*** and slight bleeding under the skin after an implant removal | After the implant or IUD has been removed for any reason and you do not want to get pregnant at that time, you should immediately start using another method of birth control, such as condoms. Women may experience slight pain and slight bleeding under the skin after an implant removal |
|  |  |  |  |
| **5. What is the best family planning method?** | **Female responses during FGD** | **Male responses during FGD** | **Revised messages with participants during FGD** |
| All modern methods of contraception prevent pregnancy. Women and their partners can choose any modern contraceptive method that is acceptable and safe for them ^26^ | All modern methods of contraception prevent pregnancy. Women and their partners can choose any modern contraceptive method that is acceptable and safe for them. | All modern methods of contraception prevent pregnancy. Women and their partners can choose any modern contraceptive method that is acceptable and safe for them. | All modern methods of contraception prevent pregnancy. Women and their partners can choose any modern contraceptive method that is acceptable and safe for them. |
| There is a wide variety of modern methods, one of which may suit you best. Condoms, when used correctly and consistently, are the only method of birth control that can prevent unwanted pregnancies and protect against sexually transmitted infections, including HIV. They can be used with other birth control methods to protect against both unwanted pregnancy and sexually transmitted infections ^26^ | There ***are many types*** of modern methods, one of which may suit you best. Condoms, when used correctly and ~~consistently~~, are the only method of birth control that can prevent unwanted pregnancies and protect against sexually transmitted infections, including HIV. They can be used with other birth control methods to protect against both unwanted pregnancy and sexually transmitted infections. | There is a wide variety of modern methods, one of which may suit you best. Condoms, when used correctly and ***when always***, are the only method of birth control that can prevent unwanted pregnancies and protect against sexually transmitted (passé passé) infections, including HIV. They can be used with other birth control methods to protect against both unwanted pregnancy and sexually transmitted infections. | There are many types of modern methods, one of which may suit you best. Condoms, when always used correctly, are the only method of birth control that can prevent unwanted pregnancies and protect against sexually transmitted infections, including HIV. They can be used with other birth control methods to protect against both unwanted pregnancy and sexually transmitted infections |
| Emergency contraceptive pills can prevent up to 95% of pregnancies when taken within five days of questionable sex (misused condom, burst condom, forgetting to take the pill or the injectable…) and they can be taken by anyone, whether or not they have a health problem. These pills do not protect against STIs and HIV-Aids ^26^ | Emergency contraceptive pills can prevent ***most of your chances of pregnancy*** when taken within five days of unprotected sex (misused condom, burst condom, forgetting to take the pill or the injectable…) and they can be taken by anyone, whether or not they have a health problem. These pills do not protect against STIs and HIV-Aids. | Emergency contraceptive pills can prevent up to 95% of pregnancies when taken within five days of unprotected sex (misused condom, burst condom, forgetting to take the pill or the injectable…) and they can be taken by anyone, whether or not they have a health problem. These pills do not protect against STIs and HIV-Aids. | Emergency contraceptive pills can prevent most of your chances of pregnancy when taken within five days of unprotected sex (misused condom, burst condom, forgetting to take the pill or the injectable…) and they can be taken by anyone, whether or not they have a health problem. These pills do not protect against STIs and HIV-Aids. |

| **- Registration of side effects** | **Female responses during FGD** | **Male responses during FGD** | **Revised messages with participants during FGD** |
| --- | --- | --- | --- |
| **Side effects that do not require intervention by a healthcare provider ^30^** |  |  |  |
| - it can wake up acne problems, | it can wake up ***pimple*** problems | it can wake up ***pimple*** problems | it can wake up pimple problems |
| - it can promote weight gain in certain predisposed women, | - it can promote weight gain in some women who are already using it, | it can promote weight gain in certain predisposed women, | - it can promote weight gain in some women who are already using it |
| - it can cause menstrual disorders (bleeding between periods, irregular periods, absence of periods), | it can cause ***period*** disorders (bleeding between periods, irregular periods, absence of periods), | it can cause ***period*** disorders (bleeding between periods, irregular periods, absence of periods), | it can cause period disorders (bleeding between periods, irregular periods, absence of periods), |
| In case of observations of other types of side effects not mentioned, it will be necessary to either, | In case you notice any other types of side effects not mentioned, it will be necessary to either, | In case you notice any other types of side effects not mentioned, it will be necessary to either, | In case you notice any other types of side effects not mentioned, it will be necessary to either, |
| -          Contact the provider | -          Contact the provider | -          Contact the provider | -          Contact the provider |
| -          Go to the nearest health facility | -          Go to the nearest health facility | -          Go to the nearest health facility | -          Go to the nearest health facility |
|  |  |  |  |
| **- Education on the different contraceptive methods record 3 ^21,29^** | **Female responses during FGD** | **Male responses during FGD** | **Revised messages with participants during FGD** |
| * **Hormonal birth control methods** include oral contraceptive pills, injectables, and implants. All prevent pregnancy ^21,29^ |  |  |  |
| - Oral contraceptive pills should be taken as advised by your healthcare professional and provide very effective protection for a 24-hour period. **^21,29^** | Oral contraceptive ***tablets*** should be taken as advised by your healthcare professional and provide very effective protection for a 24-hour period. | Oral contraceptive ***tablets*** should be taken as advised by your healthcare professional and provide very effective protection for a 24-hour period. | Oral contraceptive tablets should be taken as advised by your healthcare professional and provide very effective protection for a 24 hour period. |
| - Injectables are administered into the muscle and provide very effective protection for a period of 3 months. **^21,29^** | Injectables are administered into the ***skin*** and provide very effective protection for a period of 3 months. | Injectables are administered into the ***skin*** and provide very effective protection for a period of 3 months. | Injectables are administered into the skin and provide very effective protection for a period of 3 months. |
| - Contraceptive implants are placed under the skin of a woman's upper arm and provide very effective and continuous protection against pregnancy for a period of 3 to 5 years, depending on the type of implant. **^21,29^** | Contraceptive implants are placed under the skin of a woman's upper arm and provide very effective and continuous protection against pregnancy for a period of 3 to 5 years, depending on the type of implant. | Contraceptive implants are placed under the skin of a woman's upper arm and provide very effective and continuous protection against pregnancy for a period of 3 to 5 years, depending on the type of implant. | Contraceptive implants are placed under the skin of a woman's upper arm and provide very effective and continuous protection against pregnancy for a period of 3 to 5 years, depending on the type of implant. |
| - Emergency contraceptive pills (ECPs), taken within 5 days of unprotected sex, can prevent pregnancy. The sooner a woman takes them after sex, the more effective they are.^21,29^ | Emergency contraceptive pills (ECPs) (Postinol), taken within 5 days of unprotected sex, can prevent pregnancy. The sooner a woman takes them after sex, the more effective they are. | Emergency contraceptive pills (ECPs), taken within 5 days of unprotected sex, can prevent pregnancy. The sooner a woman takes them after sex, the more effective they are. | Emergency contraceptive pills (ECPs) (Postinol), taken within 5 days of unprotected sex, can prevent pregnancy. The sooner a woman takes them after sex, the more effective they are |
| NB: ECPs should not be used as a regular means of contraception, in place of another method. **^21,29^** | NB: ECPs should not be used ***all the time*** as a means of contraception, in place of another method. | NB: ECPs should not be used ***all the time*** as a means of contraception, in place of another method. | NB: ECPs should not be used all the time as a means of contraception, in place of another method. |
|  | **Female responses during FGD** | **Male responses during FGD** | **Revised messages with participants during FGD** |

| * **Barrier methods** are either device (male or female condom) that prevent sperm from reaching the egg, or chemicals (spermicides) that attack or destroy sperm in the vagina. They offer effective protection only for the current sexual intercourse^21,29^ | * **Barrier methods** are either device (male or female condom) that prevent sperm from reaching the egg, ~~or chemicals (spermicides)~~ that attack or destroy sperm in the vagina. They offer effective protection only for the current sexual intercourse | * **Barrier methods** are either device (male or female condom) that prevent sperm from reaching the egg, or chemicals (spermicides) that attack or destroy sperm in the vagina. They offer effective protection only for the current sexual intercourse | * **Barrier methods** are either device (male or female condom) that prevent sperm from reaching the egg, ~~or chemicals (spermicides)~~ that attack or destroy sperm in the vagina. They offer effective protection only for the current sexual intercourse |
| --- | --- | --- | --- |
| - Intrauterine devices (IUDs) are small, soft plastic devices that are placed inside the uterus. They prevent sperm from reaching the egg. Depending on their type, IUDs can provide protection for 5 to 12 years. **^21,29^** | - Intrauterine devices (IUDs) are small, soft plastic devices that are placed inside the uterus. They prevent sperm from reaching the egg. Depending on their type, IUDs can provide protection for 5 to 12 years. | - Intrauterine devices (IUDs) are small, soft plastic devices that are placed inside the uterus. They prevent sperm from reaching the egg. Depending on their type, IUDs can provide protection for 5 to 12 years. | - Intrauterine devices (IUDs) are small, soft plastic devices that are placed inside the uterus. They prevent sperm from reaching the egg. Depending on their type, IUDs can provide protection for 5 to 12 years. |
|  | **Female responses during FGD** | **Male responses during FGD** | **Revised messages with participants during FGD** |
| * **Natural methods of contraception.** |  |  |  |
| - the fertility awareness method requires the couple to know which days of the menstrual cycle the woman is fertile, ie the days when she is most likely to become pregnant. On these days, the couple must abstain from sex or use a barrier method to avoid pregnancy. **^21,29^** | the fertility awareness method requires the couple to know which days of the ***period*** the woman is safe and ***unsafe,*** ie the days when she is most likely to become pregnant. On these days, the couple must abstain from sex or use a barrier method to avoid pregnancy. | the fertility awareness method requires the couple to know which days of the p***eriod*** the woman is safe and *unsafe*, ie the days when she is most likely to become pregnant. On these days, the couple must abstain from sex or use a barrier method to avoid | the fertility awareness method requires the couple to know which days of the period the woman is safe and unsafe, ie the days when she is most likely to become pregnant. On these days, the couple must abstain from sex or use a barrier method to avoid |
| Exclusive breastfeeding has a contraceptive effect during the 6 months following childbirth if certain conditions are met. This is called the Lactational Amenorrhea Method (LAM). ^21,29^ | Feeding the child with ***only breast milk c***an provide some contraceptive effect during the 6 months following childbirth if certain conditions are met. This is called the Lactational Amenorrhea Method (LAM). | Feeding the child with ***only breast milk c***an provide some contraceptive effect during the 6 months following childbirth if certain conditions are met. This is called the Lactational Amenorrhea Method (LAM). | Feeding the child with only breast milk can provide some contraceptive effect during the 6 months following childbirth if certain conditions are met. This is called the Lactational Amenorrhea Method (LAM). |
| - The withdrawal consists for the man, as its name indicates it, to withdraw his penis from the vagina of his partner before the ejaculation in order not to deposit semen there. For most couples, the withdrawal method is one of the least effective contraceptive methods. **^21,29^** | The withdrawal consists for the man, as its name indicates it, to withdraw his penis from the vagina of his partner before the ejaculation in order not to deposit semen there. For most couples, the withdrawal method is one of the least effective contraceptive methods. | The withdrawal consists for the man, as its name indicates it, to withdraw his penis from the vagina of his partner before the ejaculation in order not to deposit semen there. For most couples, the withdrawal method is one of the least effective contraceptive methods. | The withdrawal consists for the man, as its name indicates it, to withdraw his penis from the vagina of his partner before the ejaculation in order not to deposit semen there. For most couples, the withdrawal method is one of the least effective contraceptive methods. |
|  | **Female responses during FGD** | **Male responses during FGD** | **Revised messages with participants during FGD** |
| * **Permanent methods** |  |  |  |
| - Female sterilization and male sterilization are permanent contraceptive methods. Sterilization requires relatively simple surgery and prevents lifelong pregnancy. It is an appropriate method of contraception for men and women who are sure they do not want any more children. ^21,29^ | ***Remove the womb*** and ~~male sterilization~~ are permanent contraceptive methods. Sterilization requires relatively simple surgery and prevents lifelong pregnancy. It is an appropriate method of contraception for men and women who are sure they do not want any more children. | ***Remove the womb*** and male sterilization are permanent contraceptive methods. Sterilization requires relatively simple surgery and prevents lifelong pregnancy. It is an appropriate method of contraception for men and women who are sure they do not want any more children. | Remove the womb and male sterilization are permanent contraceptive methods. Sterilization requires relatively simple surgery and prevents lifelong pregnancy. It is an appropriate method of contraception for men and women who are sure they do not want any more children. |
|  |  |  |  |
| **6. Steps to consider before using family planning** | **Female responses during FGD** | **Male responses during FGD** | **Revised messages with participants during FGD** |
| The adoption of a contraceptive method may depend on several factors: age, education, physical health, parity, profession, intervention of spouse or partner, rumour, and quality of information received on the method... ^21,29,31,32^ | The adoption of a contraceptive method may depend on several factors: age, education, physical health, parity, profession, intervention of spouse or partner, rumour, and quality of information received on the method... | The adoption of a contraceptive method may depend on several factors: age, education, physical health, parity, profession, intervention of spouse or partner, rumour, and quality of information received on the method... | The adoption of a contraceptive method may depend on several factors: age, education, physical health, parity, profession, intervention of spouse or partner, rumour, and quality of information received on the method... |
